# Phenol as proton shuttle and buffer for lithium-mediated ammonia electrosynthesis

**DOI:** 10.1038/s41467-024-46803-w

**Published:** 2024-03-18

**Authors:** Xianbiao Fu, Aoni Xu, Jakob B. Pedersen, Shaofeng Li, Rokas Sažinas, Yuanyuan Zhou, Suzanne Z. Andersen, Mattia Saccoccio, Niklas H. Deissler, Jon Bjarke Valbæk Mygind, Jakob Kibsgaard, Peter C. K. Vesborg, Jens K. Nørskov, Ib Chorkendorff

**Affiliations:** Department of Physics, Technical University of Denmark, Kongens Lyngby, Denmark

**Keywords:** Electrocatalysis, Flow chemistry, Materials for energy and catalysis

## Abstract

Ammonia is a crucial component in the production of fertilizers and various nitrogen-based compounds. Now, the lithium-mediated nitrogen reduction reaction (Li-NRR) has emerged as a promising approach for ammonia synthesis at ambient conditions. The proton shuttle plays a critical role in the proton transfer process during Li-NRR. However, the structure-activity relationship and design principles for effective proton shuttles have not yet been established in practical Li-NRR systems. Here, we propose a general procedure for verifying a true proton shuttle and established design principles for effective proton shuttles. We systematically evaluate several classes of proton shuttles in a continuous-flow reactor with hydrogen oxidation at the anode. Among the tested proton shuttles, phenol exhibits the highest Faradaic efficiency of 72 ± 3% towards ammonia, surpassing that of ethanol, which has been commonly used so far. Experimental investigations including operando isotope-labelled mass spectrometry proved the proton-shuttling capability of phenol. Further mass transport modeling sheds light on the mechanism.

## Abstract (web summary)

The proton shuttle plays a critical role in the proton transfer process during lithium-mediated ammonia synthesis. Here, the authors establish the structure-activity relationship and design principles for effective proton shuttles.

## Introduction

Ammonia (NH_3_) synthesis has shaped our modern civilization and will continue to play a key role in our planet’s future, owing to ammonia being a feedstock to produce fertilizers, polymers, pharmaceuticals, and fine chemicals^1–3^. Currently, industrial ammonia synthesis is the Haber-Bosch process using iron-based catalysts under high temperatures and pressures (i.e., 350–450 °C and 100–200 bar), which contributes ~1.3% of global carbon dioxide emissions^4^. The Haber-Bosch process demands very high pressure, and entails significant capital investment and large, centralized plants, whereas the utilization of fertilizers is characterized by decentralization. As renewable electricity prices decline, electrochemical ammonia synthesis offers the potential for decentralized fertilizer production in compact devices that can be coupled with decentralized renewable electricity sources. This development has the potential to reduce the cost of fertilizers in remote regions that lack efficient transportation networks by eliminating the need for extensive transportation^3^. Tremendous efforts have been made to achieve electrochemical ammonia synthesis by using nitrogen (N_2_) and water as feedstock and powered by renewable energy under ambient conditions^5–8^. To date, the most promising and reliable method of electrochemical ammonia synthesis is lithium-mediated nitrogen reduction reaction (Li-NRR) in nonaqueous electrolytes^9,10^. In 1930, Fichter et al. first explored the Li-NRR in an alcoholic solution of lithium halide^11^. The Li-NRR was further investigated by Tsuneto et al. using tetrahydrofuran (THF) with small amounts of ethanol (EtOH) as an electrolyte in 1993^12^. Our group proposed a rigorous procedure with gas purification and quantitative isotope measurements to avoid false positives, which validated that the produced ammonia during the Li-NRR process was from N_2_ reduction^9^. Since then, many strategies have been proposed to improve the performance of the Li-NRR system^4,13–28^. Recently, high current density (−1 A cm_geo_^−2^) and faradaic efficiency (FE) towards ammonia (close to 100%) have been achieved in a pressurized batch-type reactor (15 bar or 20 bar), but the solvent was oxidized at the anode^26,27^. Oxidation of solvent (e.g., THF and EtOH) could generate the necessary protons for ammonia production^29^. In general, most Li-NRR investigations published so far use a sacrificial solvent as a proton source^28^. In order to achieve practical viability, however, the protons must come from hydrogen (or water), so the hydrogen oxidation reaction (HOR) has been proposed as the anode reaction of the Li-NRR system to provide a sustainable hydrogen source^15,28^. Very recently, we achieved an ammonia FE of 61% in a continuous-flow reactor by employing nitrogen reduction coupled with HOR at ambient pressure and temperature^28^. Isotope-labeling studies using operando mass spectrometry revealed the hydrogen implemented in the ammonia is indeed coming from the anode via H_2_ oxidation^28^.

Generally, the Li-NRR process contains three steps to produce ammonia. Firstly, the Li^+^ is electrochemically reduced into metallic Li on the cathode and is capable of dissociating N_2_ to generate lithium surface nitride, which is protonated by a proton shuttle (e.g., EtOH) to release ammonia and Li^+^ to repeat the cycle^4,30^. The proton shuttle can have a decisive impact on the performance of the Li-NRR system. The proton shuttle screening was first investigated by Krishnamurthy et al. in the two-compartment electrochemical cell with a polyporous Daramic separator after passing 7.2 C of charge (6 min)^21^. They found that 1-butanol was the most effective proton shuttle (FE of 15.6%) and phenol (PhOH) was an inactive proton shuttle (FE of 0%). The Kamlet-Taft parameters were used as descriptors to interpret the activity toward ammonia production. Later work explained that the effect of the proton shuttle on the performance was due to the proton shuttle changing the properties of the solid-electrolyte interphase (SEI)^22^. Suryanto et al. found the phosphonium-based salts ([P_6,6,6,14_][eFAP]) can re-protonate the phosphonium cation by reacting with acetic acid but not checked in the real Li-NRR process^17^. The proton shuttles were again screened by using lithium bis(trifluoromethylsulfonyl)imide (LiTFSI) electrolyte in the batch-type reactor (15 bar)^23^. The above-mentioned works on proton shuttle screening tried to correlate the acid dissociation constant (pK_a_, values in water) of proton shuttles with the performance of the Li-NRR, but the correlation was not strong^21,23^. So far, there are no experimental studies to screen proton shuttles in the practical Li-NRR process with HOR at the anode side. Therefore, whether the proton shuttle can actually transport the protons generated by HOR to the cathode to participate in ammonia production is still unknown. Although some progress has been made, the structure-activity relationship and the role of proton shuttles in the Li-NRR process are still lacking a good understanding.

In our recent work, we successfully achieved electrochemical ammonia synthesis by coupling N_2_ reduction with HOR in the continuous-flow reactor and demonstrated the hydrogen in the produced ammonia is from HOR^28^. In this work, proton shuttle screening experiments were performed in a continuous-flow reactor in the presence of HOR at the anode after passing a charge of 700 C (over 2.5 h). We proposed a general procedure on how to prove the proton shuttle works and established design principles for effective proton shuttles in the practical Li-NRR process. Contrary to previous works^21,22^, we find that PhOH can achieve the highest FE of 72 ± 3% and an energy efficiency of 15 ± 1% at ambient pressure and temperature, which exceeds the state-of-the-art EtOH. Although the earlier work achieved an FE of almost 100% at 15 bar pressure in the batch-typed reactor, this system relies on the sacrificial agent for providing protons^26^. Theoretical mass transport modeling clarifies the dependence of the Li-NRR performance on the pK_a_ (values in THF) and diffusion coefficient of proton shuttles. Our findings offer a comprehensive framework for the rational design principles of efficient proton shuttles in Li-mediated ammonia synthesis.

## Results

### Proton shuttle screening in a continuous-flow reactor

**Fig. 1:**
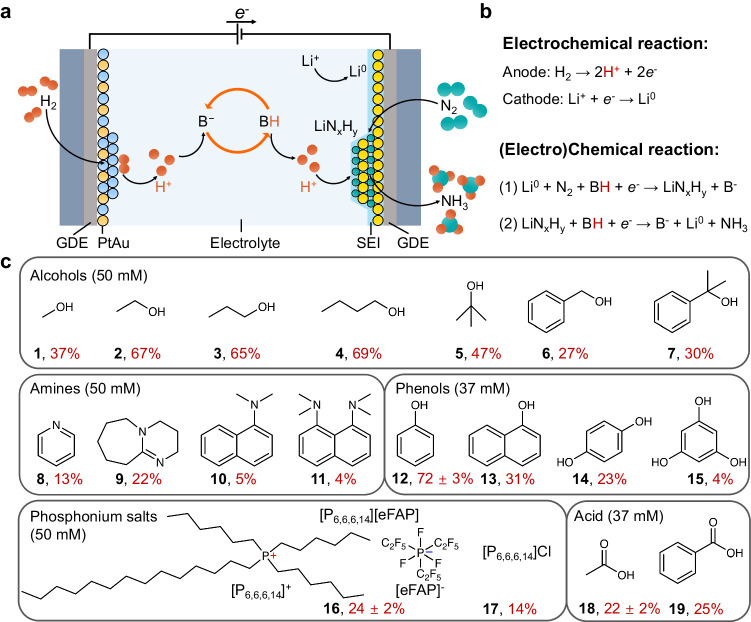
Proton shuttles screening in a continuous-flow reactor. **a** Schematic proton-shuttling process for lithium-mediated ammonia synthesis in a continuous-flow electrolyzer. **b** Lithium plating at the cathode and hydrogen oxidation reaction at the anode. **c** Ammonia faradaic efficiencies for a variety of proton shuttles. FEs towards ammonia of proton shuttles were determined by passing a total charge of 700 C (over 2.5 h) with the optimal potential cycling (−6 mA cm^−2^ for 1 min and then 0 mA cm^−2^ for 1 min) under the same test conditions. In **c** the data represent the mean ± standard deviation derived from three independent measurements.

The Li-NRR experiments are conducted in the continuous-flow electrolyzer equipped with 25 cm^2^ effective area gas diffusion electrodes (GDE) at ambient pressure and temperature (Supplementary Fig. 1). The working electrode (WE, cathode) and counter electrode (CE, anode) are 30 μm stainless steel cloth (SSC) and the PtAu/SSC, respectively (Supplementary Fig. 2). Figure 1a, shows the configuration of the continuous-flow reactor and the proton-shuttling process for lithium-mediated ammonia synthesis. The proton shuttle can participate in reactions on the formation of lithium nitride (LiN_x_H_y_) and the generation of ammonia (Fig. 1b). Protons are generated through HOR on PtAu anode catalysts^28^. These protons then react with the deprotonated form (B^−^, representing base) of the proton shuttle, resulting in the formation of the protonated shuttle (BH) of the proton shuttle. Subsequently, the protonated form diffuses to the cathode, where it protonates lithium nitride, leading to the release of ammonia and the regeneration of the deprotonated shuttle. The specific role and involved reactions of the proton shuttle in the Li-NRR process determine some requirements and design principles for efficient proton shuttles. (1) The proton shuttle should contain functional groups (e.g., -OH, -COOH, and -CHO-) or specific moiety (e.g., α hydrogen atom and -CH_2_-) that can donate/accept a proton. (2) The proton shuttle should have a proper pK_a_ in the electrolyte, which strikes a balance between protonation ability and minimizing side reactions, such as hydrogen evolution reaction (HER). If the pK_a_ of the proton shuttle is too small (i.e., highly acidic), it will undergo a direct reaction with metallic lithium, impeding N_2_ activation, or leading to the dominance of the competitive HER reaction on the cathode. Conversely, if the pK_a_ of the proton shuttle is too large (i.e., less acidic), its protonation ability will be diminished, resulting in inadequate protonation of the nitrogen atoms adsorbed on the lithium^28^. (3) The proton shuttle should possess the capacity to form a functional SEI layer on the cathode, enabling the diffusion of proton and lithium ions through this layer. For example, the EtOH plays an important role in the formation of the SEI layer^28^. (4) The deprotonated form (B^−^) of the proton shuttle should have good electrochemical stability and chemical stability. The high stability of the proton shuttle helps mitigate unwanted side reactions and ensures the overall stability of the operating system. (5) The proton shuttle should exhibit an optimal diffusion rate to effectively control the concentration of available protons on the surface of lithium nitride. Both the steric effect and hydrogen bonding can significantly impact the diffusion rate of the proton shuttle within the electrolyte. (6) The proton shuttle and the Li-NRR system should demonstrate excellent compatibility with each other. For example, the proton shuttle must not poison the HOR catalyst. Due to concerns regarding the poisoning of the anode catalysts, we refrained from testing thiols as proton shuttles in our study.

Based on these design principles for effective proton shuttles, the Li-NRR performance of various categories of proton shuttles, including alcohols, amines, phenols, phosphonium salts, and carboxylic acids, were evaluated in a continuous-flow electrolyzer under the same condition (Fig. 1c and Supplementary Table 1). Without adding proton shuttles in the electrolyte, about 60% of ammonia was generated from the electrode deposit, which implies that H_2_O, THF, and impurities in THF may serve as comparatively weaker proton shuttles. The electrolyte was 1 M lithium tetrafluoroborate (LiBF_4_) in THF with 37 mM or 50 mM proton shuttles. The ethanol (2) (bold numbers refer to Figs. 1c), 1-propanol (3), and 1-butanol (4) exhibited relatively high FE, which is consistent with previous reports^21–23^. Interestingly, a noticeable drop in FE is observed when larger groups are substituted, as evidenced by the comparison between compounds 2 and 6, as well as 5 and 7. In contrast, the amines, such as pyridine (8) and proton sponge (11), have a basic group, but cannot easily donate or abstract a proton. Therefore, amines are bad shuttles. The phosphonium salts (16, [P_6,6,6,14_][eFAP]) were synthesized by ion exchange reaction and confirmed by nuclear magnetic resonance (NMR) spectroscopy (Supplementary Fig. 3). To mitigate the influence of oxygen and water on the performance of phosphonium salts, the Li-NRR experiments were conducted within an Ar-filled glovebox^17^. The activity of phosphonium salts (16 and 17) was observed to be lower than that of alcohols, which could be attributed to the difficulty of carrying protons and lower diffusion rate (Supplementary Fig. 4). As anticipated, the acids (18 and 19) exhibited lower activity due to their highly acidic nature. Among the various proton shuttles evaluated, phenol (12) displayed the highest FE of 72 ± 3% in the Li-NRR process when used at an optimal concentration of 37 mM. This performance exceeded that of ethanol, which achieved its highest FE at an optimal concentration of 50 mM. Subsequently, more experiments and discussions will be focused on investigating phenol as a proton shuttle, considering its notable performance in the Li-NRR. It should be noted that quantitative ^15^N_2_ isotope-labeling experiments for Li-NRR have been carried out in our previous works^9,28^, thus, the isotope-labeling experiment is not shown in this work.

### Verification of phenol as a proton shuttle

**Fig. 2:**
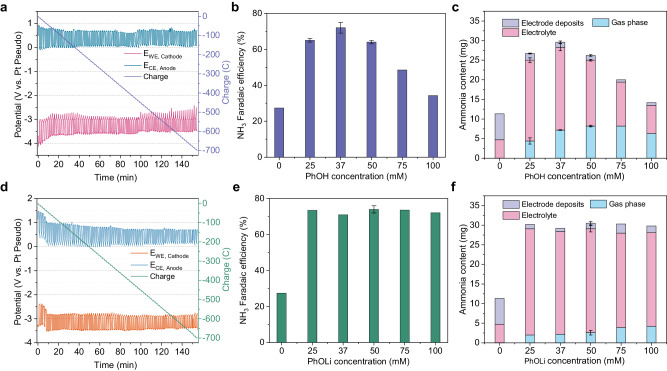
Performance of Li-mediated ammonia synthesis using phenol or lithium phenoxide as proton shuttles. **a** The chronopotentiometry using phenol as proton shuttles (37 mM). **b** Faradaic efficiency changed with varying phenol concentrations. **c** The various phenol concentrations affect the distribution of produced ammonia in the electrolyte, gas phase, and electrode deposits. **d** The chronopotentiometry using lithium phenoxide as proton shuttles (37 mM). **e** Faradaic efficiency changed with varying lithium phenoxide concentrations. **f** The various lithium phenoxide concentrations affect the distribution of produced ammonia in the electrolyte, gas phase, and electrode deposits. All potentials are without iR correction. The potential cycling condition is −6 mA cm^−2^ for 1 min and 0 mA cm^−2^ for 1 min. All experiments passed a total charge of 700 C at the current density of −6 mA cm^−2^ with the same potential cycling condition. Error bars represent the mean ± standard deviation derived from three independent measurements.

As found in our previous work, the optimal potential cycling condition entails applying a current density of −6 mA cm^−2^ for 1 min (the corresponding potential denoted deposition potential) and then 0 mA cm^−2^ for 1 min (the corresponding potential denoted resting potential)^28^. The FEs of proton shuttles were obtained by passing a total charge of 700 C under the optimal potential cycling condition. As shown in Fig. 2a, the chronopotentiometry (CP) of PhOH exhibited average anode potential and cathode potential values of approximately 0.7 V and −3.6 V versus Pt, respectively. The FE towards ammonia was found to be dependent on the concentration of PhOH, with FE of 72 ± 3% at the optimal concentration of 37 mM (Fig. 2b). This concentration dependence arises from the presence of protons within PhOH, which directly influences the available proton concentration in the Li-NRR system (Supplementary Fig. 5). In comparison to the absence of a proton shuttle, the ammonia produced using PhOH was predominantly distributed in the gas phase and electrolyte, accounting for over 90% of the total ammonia generated (Fig. 2c). During the 1 min resting period at the open-circuit voltage (OCV), the resting potential of the cathode in the CP curve is expected to increase because of the reaction between the proton shuttle and the cathode surface species. The resting potential in the absence of the proton shuttle was −3 V versus Pt, which can be the benchmark for the resting potential in the presence of the proton shuttle (Supplementary Fig. 6). For example, the resting potential with ethanol is −2 V versus Pt, but with acetate acid, it is −1 V versus Pt due to its highly acidic nature (Supplementary Figs. 7 and 8). When comparing compounds 2 and 6, 5 and 7, 18 and 19, as well as 12 and 13, a higher ratio of ammonia in electrode deposits to a total ammonia (i.e., the proton shuttle cannot protonate LiN_x_H_y_ in time and the LiN_x_H_y_ is accumulated) is observed when proton shuttles are substituted by larger groups (Supplementary Fig. 9). This higher ratio could be indicative of a lower protonation ability of the proton shuttle, which is reflected by the resting potential approaching −3 V (i.e., no oscillation) during OCV (Supplementary Figs. 6–8).

To provide further evidence of the proton shuttle’s ability to transfer protons in the actual Li-NRR process, we propose a procedure that involves utilizing the deprotonated form (B^−^) of the proton shuttle to evaluate its proton transfer capability. Additionally, when the most effective proton shuttle was identified, we suggest employing operando isotope-labeled mass spectrometry (i.e., deuterium oxidation reaction) to confirm that the hydrogen present in the produced ammonia originates from the HOR. Firstly, PhOH was substituted with lithium phenoxide (PhOLi) to evaluate its performance in the Li-NRR process. Figure 2d illustrates the CP of PhOLi, exhibiting that its anode and cathode potentials are consistent with those shown in Fig. 2a. During the initial ten minutes of CP (Fig. 2d), the anode and cathode potentials were shifted, which can be attributed to the relatively low proton concentration in the early stages. It has been proved that the gas flow composition and electrolyte composition can affect the potential stability of the Pt pseudo reference electrode^31,32^. A similar phenomenon was observed using lithium ethoxide (EtOLi) in the Li-NRR process (Supplementary Fig. 10). Unlike PhOH, the ammonia FE was independent of the concentration of PhOLi (Fig. 2e), which indicated the available proton concentration in the electrolyte was only limited by the current density of HOR. Remarkably, a FE of 74 ± 2% was achieved at a PhOLi concentration of 50 mM, which closely approached the performance achieved at the optimal concentration of PhOH. These results serve as direct evidence that the deprotonated form of PhO^−^ ions functions as the proton shuttle for proton transfer in the real Li-NRR process. When utilizing PhOLi, the distribution of ammonia in the gas phase is lower compared to using PhOH (Fig. 2f), which could be attributed to the different properties of the solid-electrolyte interphase (SEI) layer or electrode deposits.

To reveal the difference in SEI and electrode deposits between the PhOH and PhOLi (Supplementary Figs. 11 and 12), flow cell experiments were performed in an Ar-filled glovebox under four conditions — i.e., after a linear sweep voltammetry (LSV) test with either PhOH or PhOLi and after 700 C of CP with either PhOH or PhOLi. The scanning electron microscope (SEM), x-ray diffraction (XRD), and x-ray photoelectron spectrometer (XPS) transfer systems were utilized to avoid exposure to air and moisture (Supplementary Figs. 13–15). The SEM images reveal the formation of a dense SEI layer following the LSV test with PhOH, whereas fewer deposits were observed with PhOLi (Supplementary Fig. 16). After a total charge of 700 C in the CP test, the cathode surface of PhOLi exhibited an irregular and thicker layer of deposits, while a uniform and thinner layer of deposits was observed when using PhOH (Supplementary Figs. 17 and 18). In comparison to PhOH, the utilization of PhOLi as the proton shuttle exhibited a higher mass density of cathode deposits and thicker electrode deposits (Supplementary Figs. 19–21). These differences could be contributing factors to the reduced presence of ammonia in the gas phase when PhOLi was employed as the proton shuttle (Fig. 2f). The XRD patterns confirmed the main component of electrode deposits is lithium fluoride (LiF) after 700 C of the CP test with PhOH or PhOLi (Supplementary Figs. 22 and 23). The depth-profiling XPS spectra further validated that LiF was the dominant species present in both the SEI and electrode deposits when using either PhOH or PhOLi, aligning with previous findings (Supplementary Figs. 24–29)^27,28^. After 700 C of the CP test with PhOH or PhOLi, the N 1 s peak at 398 eV is attributed to LiN_x_H_y_ (Supplementary Figs. 28 and 29).

**Fig. 3:**
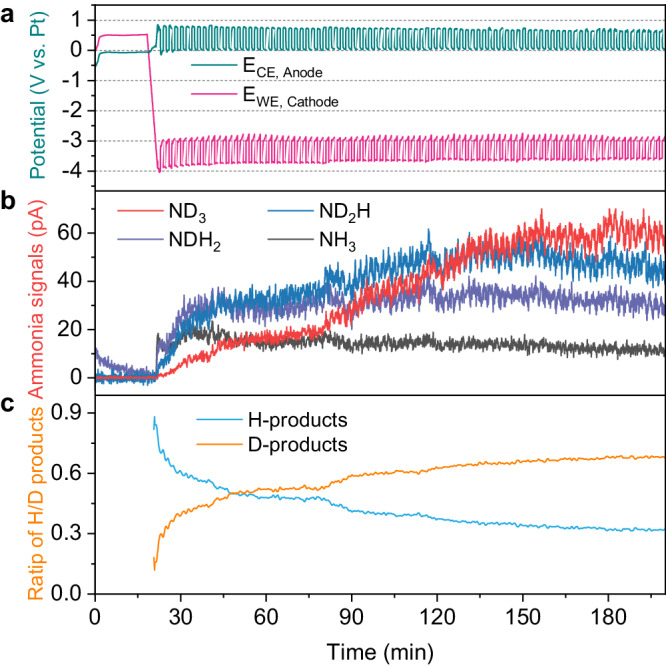
Operando mass spectrometry of cathodic ammonia gas products with isotope-labeled deuterium using phenol as proton shuttle. **a** The chronopotentiometry using phenol as proton shuttle. **b** Measured gas-phase ammonia when the anode occurs deuterium oxidation reaction. **c** The corresponding measured relative amounts of D-contained or H-contained ammonia.

Operando isotope-labeled mass spectrometry is another necessary experiment for the proposed procedure to prove the ability to transfer the proton of the proton shuttle. The operando mass spectrometry was conducted in a continuous-flow reactor, wherein a deuterium (D_2_) oxidation reaction occurred at the anode side to generate protons (D^+^) to examine whether the proton shuttle (PhOH) can transfer the protons to the cathode products (Fig. 3). The products containing deuterium (D) and hydrogen (H) were measured at the cathode side using operando mass spectrometry (Supplementary Fig. 30). Initially, the cathode is surrounded by fresh electrolyte, resulting in a predominance of H-containing products such as NH_3_ and NH_2_D (Fig. 3b). As expected, as the experiment progresses, more and more the D-containing products were generated, eventually leading to a dominance of the fully deuterated ammonia (ND_3_) (Fig. 3c). Those results unambiguously demonstrate the proton-shuttling capability of PhOH, transferring protons from HOR to the cathode products. At the initial stage, the PhOH is fully protonated with H, resulting in the generation of H-containing products. When the deprotonated form (PhO^−^) is formed, PhO^−^ can be further re-protonated by D^+^ (Supplementary Fig. 31). Subsequently, the PhOD can protonate the lithium nitrides to generate the D-containing ammonia.

**Fig. 4:**
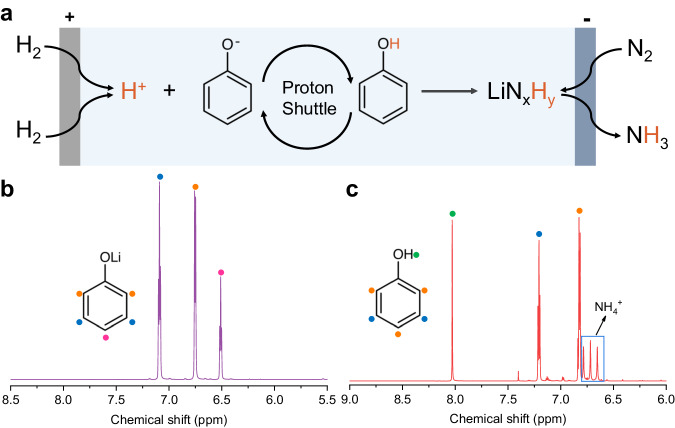
Demonstration of the ability of phenol for proton shuttling. **a** Schematic for proton-shuttling process of phenol in Li-mediated ammonia synthesis. **b** The ^1^H NMR spectra of lithium phenoxide in tetrahydrofuran before the electrochemistry test. **c** The ^1^H NMR spectra of post-reaction electrolytes using lithium phenoxide as proton shuttle after the electrochemistry test (700 C of CP).

To further elucidate the ability of the deprotonated form (PhO^−^) to interact with protons during the Li-NRR process, nuclear magnetic resonance (NMR) spectra of electrolytes were conducted before and after the electrochemistry test (Supplementary Figs. 32 and 33). As shown in Fig. 4a, the deprotonated form (PhO^−^) acts as a proton acceptor, effectively protonating the protons originating from the HOR to form PhOH. Subsequently, the PhOH reacts with LiN_x_H_y_ to release ammonia and regenerate the deprotonated form (PhO^−^). The NMR spectra of PhOLi and PhOH before the electrochemistry test are a benchmark for NMR analysis (Fig. 4b and Supplementary Fig. 34). The slight shift of ^1^H NMR peaks of PhOH before and after the electrochemistry test (Supplementary Fig. 34) was due to slight differences in the pH (i.e., after test produced ammonia). Quantitative NMR measurements indicate that the amount of PhOH remains almost unchanged after the electrochemistry test (Supplementary Fig. 35). This suggests that PhOH is relatively stable under the experimental conditions and does not undergo significant decomposition or consumption during the Li-NRR process. Notably, after the electrochemistry test, almost all of the PhOLi were observed to convert into PhOH through protonation (Fig. 4c). This conversion of PhOLi to PhOH indicates the efficient proton transfer ability of PhOLi during the Li-NRR process. Taken together, the set of experiments conducted, including the examination of the proton transfer capability of PhOLi in the real Li-NRR process, operando isotope-labeled mass spectrometry, and NMR analysis, provide unambiguous evidence of the proton-shuttling capability of PhOH.

### Deprotonated form of proton shuttle as proton buffer

Furthermore, when acetic acid was utilized as the proton shuttle, the FE of ammonia was only 22% ± 2%, primarily due to the enhanced occurrence of the competitive HER (Supplementary Fig. 36). When the mixture of PhOLi (37 mM) with acetic acid (37 mM) was employed as the proton shuttle, the FE towards ammonia improved to 31% ± 1% (Supplementary Fig. 37). Upon the addition of an equivalent amount of PhOLi, only a minor portion of PhOLi underwent transformation into PhOH (Supplementary Fig. 38). Nevertheless, a significant reduction in the HER current was observed (Supplementary Fig. 36). These results indicated the proton-buffering capability of PhOLi. Therefore, the performance improvement (about 10% FE) may be attributed to the proton-buffering capability of PhOLi (i.e., lowers the available proton concentration in the electrolyte), which effectively mitigates the adverse effects of the competitive HER (Supplementary Fig. 36).

### Stability of deprotonated form for proton shuttle

**Fig. 5:**
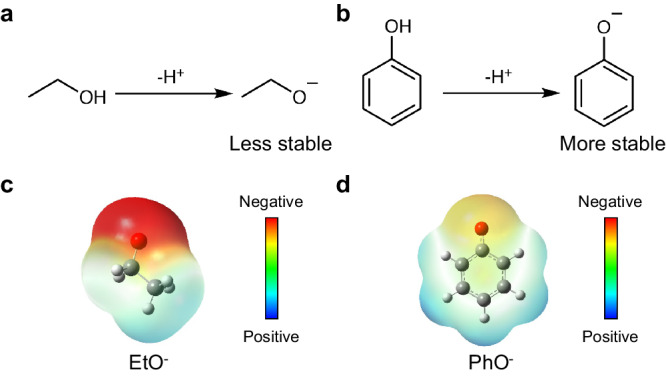
Stability of deprotonated form for proton shuttles. **a b** Deprotonated reaction of ethanol **(a)** and phenol **(b)**. **c**, **d** Electrostatic potential map of ethoxide ion **(c)** and phenoxide ion **(d)**.

One key requirement for an efficient proton shuttle is that the deprotonated form of the proton shuttle should demonstrate both excellent electrochemical stability and chemical stability during the Li-NRR process. We selected state-of-the-art EtOH as a reference for comparing stability. Figure 5a, b illustrate the deprotonation reactions of EtOH and PhOH, respectively. It is well known that the resonance-stabilized phenoxide ion is more stable than an ethoxide ion^33,34^, evidenced by the electrostatic potential maps from density functional theory (DFT) calculations (Fig. 5c,d), where less negative charge resides on the oxygen atom of phenoxide, compared to ethoxide. When lithium ethoxide (EtOLi) was used as a proton shuttle, the FE achieved was only 26 ± 1% (Supplementary Fig. 39), significantly lower than that of PhOLi (74 ± 2%). The lower performance of EtOLi may be attributed to the relatively lower stability of the ethoxide ion compared to the phenoxide ion (Supplementary Figs. 35 and 40). The electrochemical stability of EtOH and PhOH was investigated in an aqueous electrolyte (Supplementary Fig. 41). The onset and peak oxidation potentials revealed a distinct stability order under the same experimental conditions: PhOH demonstrated superior stability compared to EtOH and THF, a result consistent with previous studies^35–37^. Notably, the oxidation current density of PhOH was significantly smaller than that of EtOH, indicating excellent electrochemical stability. These findings emphasize the stability of PhOH as an efficient proton shuttle in the practical Li-NRR process.

**Fig. 6:**
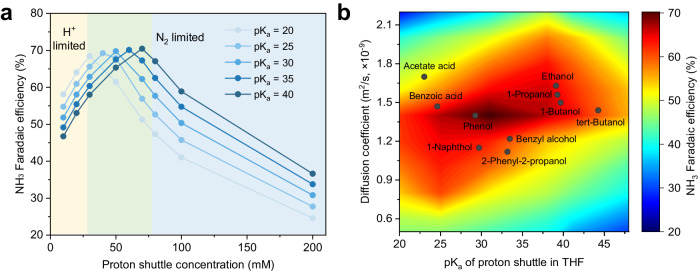
Dependence of the Li-NRR performance on the pK_a_ and diffusion coefficient of proton shuttles. **a** A 1D plot of ammonia faradaic efficiency versus concentrations of proton shuttle with different pK_a_. **b** Heatmap of the predicted ammonia faradaic efficiency as a function of the pK_a_ of proton shuttle (x-axis) and diffusion coefficient (y-axis).

Thermodynamic calculations indicate that, on the cathodic side, all electrochemical steps of NRR are energetically favorable under operation^30^, while HOR is highly selective on the PtAu anode depending on whether ethanol or phenol is the shuttle (Supplementary Fig. 42). We, therefore, involve HOR as anodic reaction into a mass transport model, on the basis of our previous atomic-kinetic model^14^, by a direct conversion between current density and proton flux from HOR (details see “Computational methods” section). We then employed a mass transport model to simulate the Li-NRR system based on the fact that diffusion of N_2_ and H^+^ is significantly slower than reaction kinetics and rate-limiting overall. The model, which is based on the model and extended to include homogeneous reactions in electrolytes^14^, is described in detail in “Computational methods”, showing general consistency with experimental data (Supplementary Fig. 43)^4,28^, contributing to a reasonable understanding of the volcano-like relationship for proton shuttle concentrations vs. NH_3_ selectivity, and a prediction of shifted trend of optimal FE at the balance between N_2_ vs. H^+^ flux. The latter is dependent on proton shuttle concentrations and species. In the case of acidic shuttles with a lower pK_a_, e.g., acetate acid, the proton is much easier to transport from anode to cathode by protonation and deprotonation, leading to a larger value of proton flux at the same concentration, thereby an increased FE of ammonia in the H^+^-limited region and a decreased FE in the N_2_-limited region, as shown in Fig. 6a. The deprotonated form (B^−^), unable to contribute to proton concentration in electrolytes, could retain the optimal performance when further increasing concentration (Supplementary Fig. 44), agreeing with experimental observation (Fig. 2e). At a specific proton shuttle concentration like our experimental setup, a new volcano plot (Supplementary Fig. 45) would be able to elucidate the effects of proton shuttle pK_a_.

Accurate evaluation of pK_a_ in THF is essential to quantify proton shuttle influence. We applied solvent-corrected DFT to benchmark pK_a_ calculation (Supplementary Fig. 46) for a series of alcohols and acids. The calculation outcomes (Supplementary Table 2) show good agreement with the literature and expand the capacity of pK_a_ datasets in organic solvent^38^. Taking the calculated pK_a_ into the mass transport model, we found that the single descriptor, pK_a_, cannot completely explain proton shuttles’ influence on Li-NRR (Supplementary Fig. 47). As an example, an exception is phenol and 1-naphthol which have very close pK_a_ values (29.24 vs. 29.70), but significant difference in NRR FE performance (72% vs. 30%). Considering the size difference between phenol and 1-naphthol, one may posit the size-related diffusivity of the proton shuttle, as the second descriptor for catalytic performance. At a specific pK_a_ value, a proton shuttle with low diffusivity would, at the same time, have slower proton diffusion, requiring compensation from increasing its concentration (Supplementary Fig. 48). The synergistic effects of pK_a_ and diffusivity on the resulting FE are graphically represented by Fig. 6b, in which an optimal NH_3_ FE would require either acidic but transport-limited proton shuttles or basic but transport-fast shuttles, in agreement with experimental discovery. The trade-off between diffusion and pK_a_ is a materialization of H^+^ flux, still aligning with our original mass transport model^14^.

In summary, proton shuttle screening has been performed in the practical Li-NRR process with HOR at the anode in a continuous-flow reactor. Proton shuttles play a crucial role in the transfer of protons from the HOR at the anode to the cathode for ammonia synthesis in the Li-NRR process. We propose a general procedure to validate the effectiveness of proton shuttles in the Li-NRR and establish design principles for developing efficient proton shuttles in practical Li-NRR processes. Through this procedure, we demonstrate that the PhOH exhibited excellent proton transfer capabilities and stability in the Li-NRR process. Moreover, the PhOH exhibited an ammonia FE as high as 72 ± 3%, which is among the highest ammonia selectivity achieved in Li-NRR at ambient conditions. The deprotonated form (PhO^−^) of the PhOH is proven to act as the primary species responsible for transferring protons during the Li-NRR process. The combination of theoretical calculations and micro-kinetic modeling provides an understanding of the mechanism of proton shuttling in the Li-NRR process. The findings contribute to the understanding of the mechanistic aspects and design principles for efficient proton shuttles in practical Li-NRR applications, ultimately paving the way for the development of sustainable and environmentally friendly ammonia production methods.

## Methods

### Materials

All solvents and reagents were used as received without further purification unless otherwise specified. 316 stainless steel cloth (McMaster-Carr, 500 × 500 mesh, pore size of 30 μm), 316 stainless steel cloth (McMaster-Carr, 325 × 2300 mesh, pore size of 5 μm), Pt wire (Goodfellow, 99.99%, diameter: 0.5 mm), H_2_PtCl_6_·6H_2_O (Sigma–Aldrich, ACS reagent), HAuCl_4_·3H_2_O (Sigma–Aldrich, 99%), H_2_SO_4_ (Sigma–Aldrich, 99.999%), tetrahydrofuran (THF, Sigma–Aldrich, anhydrous, >99.9%, inhibitor-free), LiBF_4_ (Sigma–Aldrich, ≥98%, anhydrous), ethanol (EtOH, Honeywell, anhydrous), lithium ethoxide (EtOLi, Sigma–Aldrich, 95%), methanol (Sigma–Aldrich, ≥99.9%), 1-propanol (Sigma–Aldrich, ≥99.9%), 1-butanol (Sigma–Aldrich, 99.9%), tert-butanol (Sigma–Aldrich, anhydrous, ≥99.5%), benzyl alcohol (Sigma–Aldrich, anhydrous, 99.8%), 2-phenyl-2-propanol (Sigma–Aldrich, 97%), pyridine (Sigma–Aldrich, anhydrous, 99.8%), 1,8-diazabicyclo[5.4.0]undec-7-ene (DBU, Sigma–Aldrich, ≥99%), N,N-dimethyl-1-naphthylamine (NDN, Sigma–Aldrich, ≥ 98%), N,N,N′,N′-tetramethyl-1,8-naphthalenediamine (proton sponge, Sigma–Aldrich, 99%), phenol (PhOH, Sigma–Aldrich, ≥99.0%), 1 M lithium phenoxide in THF solution (PhOLi, light yellow, Sigma–Aldrich), 1-naphthol (Sigma–Aldrich, ≥99%), hydroquinone (Sigma–Aldrich, ≥99%), phloroglucinol (Sigma–Aldrich, ≥99%), acetate acid (Sigma–Aldrich, ≥99.99%), benzoic acid (Sigma–Aldrich, ≥99.5%), trihexyltetradecylphosphonium chloride (Sigma–Aldrich, ≥95%), 1-ethyl-3-methylpyrrolidinium tris(pentafluoroethyl)trifluorophosphate (Sigma–Aldrich, high purity), dichloromethane (DCM, Sigma–Aldrich, ≥99.8%).

### Synthesis procedures of phosphonium salts (Trihexyltetradecylphosphonium tris(pentafluoroethyl)trifluorophosphate, [P_6,6,6,14_][eFAP])

10.4 g of 1-ethyl-3-methylpyrrolidinium tris(pentafluoroethyl)trifluorophosphate (20 mmol, M = 519.3 g/mol) and 11.1 g of trihexyltetradecylphosphonium chloride (20 mmol, M = 556 g/mol) were added to a 1:1 mixture of water (H_2_O) and dichloromethane (DCM). The mixture was stirred vigorously for 1 h at room temperature. After the reaction, the organic DCM solution was washed with miliQ H_2_O approximately 10 times until the silver chloride test showed no reaction. After purification, DCM was removed in a vacuum at 40 °C on a rotary evaporator. The residual water was removed by dissolving the resulting material in benzene and distilling it under a vacuum at 50 °C. After purification, the resulting slightly yellowish compound was left drying in the oven at 120 °C for at least overnight or over the weekend. The ^1^H, ^31^P, and ^19^F NMR showed a pure product and no starting material or contamination. The yield of the reaction was 97%.

### Material characterization

X-ray diffraction (XRD), X-ray Photoelectron Spectroscopy (XPS), and scanning electron microscope (SEM) were used to characterize electrodes and cathode electrode deposits. To analyze the solid-electrolyte interface (SEI, after LSV) or post-reaction electrodes (electrode deposits, after 700 C), WE electrodes were characterized after electrochemistry with XRD, XPS, and SEM. The flow cell experiments were performed in an Ar-filled glovebox and a transfer system was used to minimize the exposure to air. For XRD, a gas-tight polycarbonate (PC) dome (Anton Paar) was used to cover the sample on top of the XRD sample holder during the transport from the glovebox to the instrument as well as during the measurement. For XPS, the samples were transported from the glovebox to the XPS via a vacuum transfer system. The SEM samples were transported from the glovebox to the SEM via a cryo-SEM transfer rod. XRD data were recorded with the Data Collector v5.4 software on a Malvern Pananalytical Empyrean X-ray diffractometer. On the incident beam path, a parallel beam X-ray mirror for Cu radiation was used with a fixed 10 mm mask and a 1/8° slit placed 140 mm from the sample. The diffracted beam path was equipped with a parallel plate collimator having an opening of 0.18°. The source was an Empyrean Cu LFF HR gun operated at 45 kV and 40 mA, with Kα_1_ = 1.540598 Å and a Ni beta-filter. The data were analyzed with HighScore Plus v4.6a by Panalytical with reference patterns from the Inorganic Crystal Structure Database (ICSD). X-ray Photoelectron Spectroscopy was performed using a ThermoScientific Thetaprobe instrument equipped with an Al Kα X-ray source with a chamber base pressure below 9·10^−10^ mbar. Survey spectra were recorded with 50 scans at 50 ms dwell time per 1 eV step. Elemental detail spectra were recorded with 25–100 scans in 0.1 eV steps with 50 ms dwell time. The lateral resolution was 400 μm and a value of pass energy of 100 or 200 eV was used. The chamber pressure was risen to 1.3·10^−7^ mbar by flowing 6.0 Ar gas as required using a flood gun, which was used in charge neutralization mode during the measurement. The data were acquired and analyzed using Thermo Advantage v5.9917 by Thermo Fischer Scientific. All data were fitted with the Powell fitting algorithm and the background was determined by the Smart background option, which is based on the Shirley background.

### Preparation of cathode electrodes

The 316 stainless steel cloth (SSC, McMaster-Carr, 500 × 500 mesh, pore size of 30 μm) was used as the cathode electrodes for the electrochemical ammonia synthesis test. Before use, the SSC was cleaned with acetone and ethanol 3 times. The treated SSC was dried in the vacuum oven for further use.

### Preparation of anode electrodes

The PtAu/SSC was prepared by electrodeposition as previously described. Brief description for the electrodeposition, SSC with a pore size of 5 μm (McMaster-Carr, 325 × 2300 mesh) was used as the working electrode, and two Pt mesh electrodes (Goodfellow, 1.5 cm × 1.5 cm, 99.9%) were electrically connected and used as a split counter electrode. The 10 mM H_2_PtCl_6_·6H_2_O (Sigma–Aldrich, ACS reagent) with 10 mM HAuCl_4_·3H_2_O (Sigma–Aldrich, 99%) in 3 M H_2_SO_4_ (Sigma–Aldrich, 99.999%) solution was used as the electrolyte for electrodepositing PtAu/SSC. A current density of −0.2 A/cm^2^ was applied for 2 min in which rigorous hydrogen evolution and metal deposition took place at the same time, leading to high surface area structures of PtAu on the SSC. After the electrodeposition, the electrodes were rinsed in EtOH and ultra-pure water (18.2 MΩ resistivity, Millipore, Synergy UV system) several times to remove residual electrolytes.

### Electrochemical experiments

The electrochemical ammonia synthesis was performed in a three-chamber flow cell (Supplementary Fig. 1). The effective electrode area of the flow cell was 25 cm^2^. The central electrolyte chamber is made of polyetheretherketone (PEEK, THF-resistant materials). The N_2_ (5.0, Air Liquide) and H_2_ (5.0, Air Liquide) gas flow rates were controlled using a mass flow controller (Brooks Instrument) and set to 75 sccm. The N_2_ and H_2_ used in the experiments were cleaned by purifiers (NuPure) to reduce labile N-containing compounds to parts per trillion by volume (ppt-v) level. Before each experiment, the flow cell parts were boiled in ultra-pure water for 3 h and dried overnight at 100 °C in an oven. The cleaned SSC (500 × 500 mesh, thickness: 30 μm) was used as the working electrode (WE, cathode). As-prepared PtAu/SSC was used as the counter electrode (CE, anode). The pseudo reference electrode (RE) was a Pt wire (Goodfellow, 99.99%, diameter: 0.5 mm). Before electrochemical tests, the Pt wire was flame-annealed.

The LiBF_4_ (Sigma–Aldrich, ≥98%, anhydrous) was dried at 120 °C for 48 h in a vacuum oven before use. Ethanol (Honeywell, anhydrous) was dried with 3 Å molecular sieves. Electrolyte solution consisted of 1.0 M LiBF_4_ in tetrahydrofuran (THF, anhydrous, >99.9%, inhibitor-free, Sigma–Aldrich) and 0–100 mM proton shuttles and was prepared in an argon-filled glovebox. A syringe pump (World Instruments) was used to control the flow rate of the electrolytes at 1.0 ml/min. The syringe (Trajan Scientific and Medical, 100 ml) consisted of a borosilicate glass tube and a PTFE plunger (Gas tight). 1 mM of HCl (Sigma–Aldrich, Suprapur) aqueous solution was used to control the back pressure and trap the gas-phase ammonia. The gas outlet back pressure of the flow cell was modulated by a 10.5 centimeters 1 mM of HCl solution column (50 mL), which leads to a pressure gradient of 15 mbar between the gas inlet and outlet of the flow cell. Before the injection of electrolyte into the electrolyte chamber, the purified N_2_ and H_2_ (75 sccm) were introduced into the empty assembled flow cell for at least 30 min. Afterward, the electrolyte solution was injected into the cell in N_2_ and H_2_ atmospheres.

The electrochemistry experiments were conducted using a BioLogic Potentiostat (VMP2). The resistance between the WE and RE was measured using the potentiostatic electrochemical impedance spectroscopy (PEIS) and the current interrupt technique. The linear sweep voltammetry (LSV) was recorded from the open-circuit voltage (OCV) until lithium plating is clearly seen. Subsequently, chronopotentiometry (CP) was measured with potential cycling. The potential cycling refers to that −6 mA/cm^2^ (the corresponding potential denoted deposition potential) was applied for 1 min and then 0 mA/cm^2^ (the corresponding potential denoted resting potential) for 1 min. 700 C of charge was passed for each experiment to determine the ammonia Faradaic efficiency (FE). We note that all experiments were conducted at room temperature and 1 bar pressure. Typically, flow cell experiments were performed in a fume hood. For analysis of the electrode deposits on the working electrode, the flow cell was run in an Ar-filled glovebox. As the phosphonium salts are sensitive to the water content, using phosphonium salts as proton shuttle experiments were run in an Ar-filled glovebox. The water content of the electrolyte was measured by Karl Fischer Titration (831 KF Coulometer and 728 Stirrer, Metrohm). Before the electrochemistry test, the water content in the electrolyte with phosphonium salts is about 15–21 ppm. When the experiment finished, total NH_3_ production was quantified from the (1) gas-phase NH_3_ trapped in 1 mM of HCl, (2) NH_3_ trapped in the electrolyte, and (3) NH_3_ trapped in the electrode deposits. Typically, 120–130 ml of ultra-pure water was used to dissolve the electrode deposits to release trapped NH_3_. We note that H_2_O can react with N-containing compounds (e.g., LiN_x_H_y_) in the electrode deposits to produce NH_3_.

### THF, EtOH, and PhOH oxidation experiments in aqueous electrolyte

A good proton shuttle should have high electrochemical stability during the Li-RR process. To evaluate the electrochemical stability of THF, EtOH, and PhOH on the PtAu anode catalysts, the oxidation experiments in an aqueous electrolyte were carried out in a custom-made three-electrode glass cell using the rotating disk electrode method. The Hg/Hg_2_SO_4_ reference electrode was calibrated before the electrochemistry test. A Pt wire was used as a counter electrode. The working electrode (PtAu/Ti) was obtained from electrodeposited PtAu on a titanium stub (ϕ = 5 mm, 0.196 cm^2^). To prepare the PtAu/Ti electrode, an electrolyte of 10 mM H_2_PtCl_6_·6H_2_O (Sigma–Aldrich, ACS reagent) with 10 mM HAuCl_4_·3H_2_O (Sigma–Aldrich, 99%) in 3 M H_2_SO_4_ (Sigma–Aldrich, 99.999%) was used. A current density of −1 A/cm^2^ was applied for 2 min in which rigorous hydrogen evolution and metal deposition took place at the same time with 2500 revolutions per minute. The cyclic voltammetry (CV) curve was recorded between 0 and 1.2 V vs RHE at 50 mV/s in Ar-saturated 0.1 M H_2_SO_4_. The THF, EtOH, and PhOH oxidation activity was evaluated in Ar-saturated 0.1 M H_2_SO_4_ with 0.1 M THF or 0.1 M EtOH or 0.1 M PhOH electrolyte with a scan rate of 50 mV/s.

### D_2_ oxidation experiment via online mass spectrometry

Mass spectrometry (MS) measurements were collected with a QMG 422, using a 100 mm long QMA 120 quadrupole mass filter (QMS) from Balzers. The QMS was optimized for soft ionization measurements at 26 eV ionization energy, ensuring minimum cracking patterns in the mass spectra, while allowing for a decent signal. The experimental setup is schematically shown in Supplementary Fig. 30. The MS is placed downstream on the N_2_ flow from the cathode side of the flow cell. This allows for operando measurements of the flow cell operation and performance. The connecting gas line is heat traced to 100 °C, to avoid sticking ammonia to the inner tube walls and increase collection efficiency and time resolution. The continuous gas collection is through a 1 µm flow calibrated orifice from Lenox Laser. Measurements with electrolytes in the flow cell are collected under a low vacuum of typically 2.4 × 10^−6^ mbar, while the vacuum chamber is heated to 120 °C. The vacuum is sustained by a HiPace 300 H Turbo Pump from Pfeiffer Vacuum. The electrochemical reaction was performed in the flow cell electrolyzer. The electrolyte consisted of 1.0 M LiBF_4_ in THF with 37 mM phenol. The electrochemical test is similar to the previous section. Deuterium (D_2_, 99.8 atom % D, 4.5 N purity) and nitrogen (N_2_, 5 N purity) flow rates were controlled with mass flow controllers (Brooks), and both were set to 50 ml/min. The liquid electrolyte was continuously flown at ambient pressure at 1 ml/min, controlled by a peristaltic pump with PTFE-lined tubing (ZHUNZE, BF400H). Gas pressure was modulated by passing outlet gas through a 10.5 cm acidified water column. D_2_ outlet gas was passed directly through a water column, whereas the N_2_ gas outlet was flowed by the mass spectrometer before passing through a water column. Once both gasses and electrolytes were flowing, the mass spectrometer was started and measured on the N_2_ gas outlet. The baseline mass signals were measured during OCV, after which the potential cycling procedure was started, switching between 1 min deposition at −6 mA/cm^2^ and 1 min resting at 0 mA/cm^2^.

### Quantification of ammonia

Ammonia production was quantified using ion chromatography (IC, Metrohm) with an autosampler. The materials of the connection tubes, pressure screws, capillaries, and injection needles were PEEK or PTFE (THF-resistant materials). The IC could measure THF-containing samples that were diluted with ultra-pure water. The IC detection system is a conductivity detector. The cation column (Metrosep C6) was used as a high-capacity separation column. The Metrosep C6 column material was silica gel with carboxyl groups. The eluent (mobile phase) was 3.3 mmol/L HNO_3_ (Sigma–Aldrich, Suprapur) solution with 10 vol.% acetone (Sigma–Aldrich, ≥99.9%). The eluent flow rate was 0.9 ml/min. Before use, the eluent was degassed for 30 min to prevent gas bubbles in the high-pressure pump. For the IC measurements, a 20 μl volume of the sample was injected and measured for 20 min. As THF-containing electrolytes could damage the IC and a high concentration of calcium ions could cause adsorption saturation of the column, the electrolyte was diluted 10–100 times with ultra-pure water. The dilution factor depends on the specific experiment and the expected amount of ammonia produced. To protect the cation column, if the sample or diluted sample contains visible particles, it was pretreated by a filter syringe with a PTFE filter membrane (Whatman Puradisc, 0.45 μm) to remove the particles before testing IC. Based on the IC settings and conditions, the retention time for the standard cation solution (Sigma–Aldrich) was: 6.48 min for Li^+^, 8.96 min for Na^+^, and 15.32 min for K^+^. The retention time of NH_4_^+^ was 10.60 min. Ammonium chloride (Sigma–Aldrich, 99.998%) solution was used to make the NH_3_ calibration curve. The reliability of the IC method was verified by the colorimetric indophenol method, previously described^28^.

### Nuclear magnetic resonance (NMR) Analysis

The NMR characterization of the samples was performed at 25 °C using a Bruker AVANCE III HD spectrometer operating at ^1^H frequency of 800 MHz equipped with a 5 mm TCI CryoProbe (Bruker Biospin). The samples contained THF with 2 signals in the spectra at approximately 2 and 4 ppm which were reduced to extract the signals of interest. The spectra also contained 2 signals of ethanol at approximately 1.5 and 4 ppm, however, the proton on the oxygen is drifting and might not be seen in general in the spectrum. The chemical shifts were normalized to tetramethylsilane (TMS). In general, some variation in the chemical shift of the NMR signals could appear due to some differences in the pH, volume, and/or temperature of the sample. The data were analyzed with Bruker Topspin 4.1.4 with an academic license. 37 mM of phenol in THF and 50 mM of lithium phenoxide in THF were tested as a reference. For the phosphonium salt ([P_66614_][eFAP]), the ^1^H, ^31^P, ^15^N, ^13^C and ^19^F NMR were recorded. For quantitative analysis, the signal of acetone (270 mM) which was used as a calibration compound was integrated and normalized according to the number of protons giving rise to the signal. In all spectra, there was only one signal of acetone at ~2.3 ppm meaning that no enol tautomer was present. The concentration of a compound of interest (x) in the presence of acetone as a calibrant was calculated according to the formula *C*_x_ = *I*_x_/*I*_cal_ × *N*_cal_/*N*_x_ × *C*_cal_ where I, N, and C are the integrated area, number of nuclei, and concentration of the compound of interest (x) and the calibrant (cal), respectively. It is important to mention that the assumption is the acetone is totally pure.

### Faradaic efficiency calculations

To calculate the Faradaic efficiency (FE), the concentrations of synthesized ammonia in the electrolyte (*c*_*1*_), gas-phase trapped solution (*c*_*2*_), and electrode deposits dissolved solution (*c*_*3*_) were measured via ion chromatography, along with the volume, V_1_, V_2_, and V_3_, respectively, after each measurement. This is compared with the total charged passed, Q (unless otherwise specified, it is 700 C):

$${Q}_{{{{{{\rm{N}}}}}}{{{{{{\rm{H}}}}}}}_{3}}=3\cdot F\cdot ({c}_{1}\cdot {V}_{1}+{c}_{2}\cdot {V}_{2}+{c}_{3}\cdot {V}_{3})$$

$$F{E}_{N{H}_{3}}=\frac{{Q}_{{{{{{\rm{N}}}}}}{{{{{{\rm{H}}}}}}}_{3}}}{Q}$$

where F is Faraday’s constant, and 3 is the number of electrons transferred during the reaction for each mole of NH_3_.

### Determination of the ammonia content in electrolyte deposits

After the electrochemical test, the cathode electrode (containing the electrolyte deposits) was immersed in ultra-pure water (120–130 ml) and ultrasonicated for 2 min. The electrode deposit dissolved solution was pretreated by a filter syringe with a PTFE filter membrane (Whatman Puradisc, 0.45 μm) to remove the particles. After that, the filtered solution was measured via ion chromatography (IC). Based on this, the released ammonia of the electrode deposit was determined. It should be noted that H_2_O can react with N-containing compounds (e.g., LiN_x_H_y_) in the electrode deposits to release NH_3_. With effective proton shuttles in the electrolyte, the produced NH_3_ from electrode deposits should be in a small amount. The amount of ammonia released from the electrode deposits can be used as an indicator of the protonation ability of the proton shuttle. In other words, the more ammonia released from the electrode deposits, which means the lower the protonation ability of the proton shuttles.

#### Computational methods

##### Reaction energies calculations

Density functional theory (DFT) calculations were employed to calculate reaction energies for HOR and ethanol, phenol oxidation on PtAu catalysts, with the Vienna Ab Initio Simulation Package (VASP) code^39,40^. Based on our previous work, here we took PtAu(211), 8-Pt aggregate PtAu(211), and Pt/Au(111) facets into consideration. Ethanol oxidation was simulated in a pathway of CH_3_CH_2_OH → CH_3_CH_2_O → CH_3_CHO → CH_3_CO → CH_2_CO → CH_2_-CO, while phenol oxidation along C_6_H_5_OH → C_6_H_5_O. In all calculations, the exchange-correlation energy was modeled by using Perdew-Burke-Ernzerhof (PBE) functional within the generalized gradient approximation (GGA)^41^. The projector-augmented wave (PAW) pseudo-potentials were used to describe ionic cores^42^. The cutoff energy of 750 eV was adopted for all adsorption calculations. A Methfessel-Paxton smearing of 0.05 eV to the orbital occupation was applied during the geometry optimization and for the total energy computations. In all calculations, the atoms at all positions have Hellmann–Feynman forces lower than 0.05 eV Å-1 and the electronic iterations convergence was 10^−5^ eV using the Normal algorithm. All configurations can be found in the public GitHub repository: https://github.com/onealshu/phenol.git. During the adsorption calculations, the bottom two layers are fixed at the tested lattice positions while other layers including adsorbates are fully relaxed. Reaction energies were calculated based on the computational hydrogen electrode model^43^.

##### The pK_a_ calculations

The pK_a_ for a molecule was calculated from the solution phase free energy of the deprotonation reaction,

$${BH}\left({solv}\right)\mathop{\to }\limits^{{\triangle G}_{{solv}}}{B}^{-}\left({solv}\right)+{H}^{+}\left({solv}\right)$$

$${{pK}}_{a}=\frac{{\triangle G}_{{solv}}}{2.303{RT}}$$

where ${\triangle G}_{{solv}}={G}_{{solv}}\left({{B}}^{-}\right)+{G}_{{solv}}\left({H}^{+}\right)-{G}_{{solv}}\left({BH}\right)$. Free energies *G*_*solv*_ were obtained by considering electronic energies and frequency corrections, in the sum of electronic and thermal free energies from Gaussian 16^44^. Atomic models are established in explicit THF molecules, after benchmarking the number of THF (Supplementary Fig. 46). During calculations, all atoms are flexible in three directions and considered to compute frequency. Relaxations and frequency calculations were performed with functional of B3LYP^45^, 6–31 + + G(d,p) basis set, at *P* = 101,325 Pa and *T* = 298.15 K. For ions like proton or ${{B}}^{-}$, extra positive/negative charge was set in the explicit model. All configurations can be found in the same GitHub repository.

##### Mass transport simulations

A 1-D full mass transport limiting model was developed to simulate the Li-NRR system. Nernst-Planck equation was employed to simulate the diffusion and migration of species ${c}_{i}$ in the model, with average conductivities $\sigma$ for the SEI layer and boundary layer (liquid electrolyte).

Planck equation with a boundary thickness of 12 µm.

$$\frac{\partial {c}_{i}}{\partial t}=\nabla \left({D}_{i}\nabla {c}_{i}+\frac{{{z}_{i}D}_{i}}{{RT}}F{c}_{i}\nabla {\varphi }_{l}\right)$$

$$\sigma \nabla {\varphi }_{l}=-F\left(\sum {z}_{i}\frac{\partial {c}_{i}}{\partial t}\right)$$

where *c* is the concentration, *D* is the diffusion coefficient, *z*_*i*_ is the charge of species i, ${\varphi }_{l}$ is the potential of the electrolyte, *t* is the time, *R* is the ideal gas constant and *T* is the temperature, $F$ is the Faraday constant.

To solve these equations, we set up the Neumann boundary conditions for NH_3_ and H_2_, while Dirichlet boundary conditions for N_2_ and H^+^ to simulate the full mass transport limiting condition at the electrode side:

x = 0 (electrode surface):

$${f}_{{{NH}}_{3}}=\left\{\begin{array}{c} \! \! \! 2{f}_{{N}_{2}},\quad {f}_{{N}_{2}} < \frac{1}{6}{f}_{{H}^{+}}\\ \frac{1}{3}{f}_{{H}^{+}},\quad {f}_{{N}_{2}}\ge \frac{1}{6}{f}_{{H}^{+}}\end{array}\right.$$

$${f}_{{H}_{2}}=\left\{\begin{array}{c}\frac{1}{2}({f}_{{H}^{+}}-{6f}_{{N}_{2}}),\quad\quad {f}_{{N}_{2}} < \frac{1}{6}{f}_{{H}^{+}}\\ 0,\hfill \quad\quad\quad {f}_{{N}_{2}}\ge \frac{1}{6}{f}_{{H}^{+}}\end{array}\right.$$

$${c}_{{H}^{+}}^{{surf}}=0$$

$${c}_{{N}_{2}}^{{surf}}=0$$

To simulate the influence of anodic reaction, we involve HOR in the model by setting a constant boundary condition for proton flux, ${f}_{{H}^{+}}$, at the end of the boundary layer,

$${f}_{{H}^{+}} \,=\, \frac{{j}_{{HOR}}}{F}$$

*F* is Faraday constant.

Protonation and deprotonation reactions of proton shuttle ${BH}$ like ethanol or phenol were considered in the boundary layer,

$${BH}\begin{array}{c}{k}_{+}\\ \rightleftharpoons \\ {k}_{-}\end{array}{B}^{-}+{H}^{+}$$

All parameters and set up in the model can be found in the same GitHub repository.

### Supplementary information

Supplementary Information

Peer Review File

## Data Availability

All data reported in the main text and Supplementary Information are available from the corresponding author upon reasonable request.
