# Supplementary material for: Phenol as proton shuttle and buffer for lithium-mediated ammonia electrosynthesis
Source: Nat Commun. 2024 Mar 18;15:2417. doi: 10.1038/s41467-024-46803-w (PMC10948763; doi:10.1038/s41467-024-46803-w)
Supplement: Supplementary file 1 — Supplementary Information [file 41467_2024_46803_MOESM1_ESM.pdf]

Supplementary Information

Phenol as Proton Shuttle and Buffer for Lithium-mediated Ammonia Electrosynthesis

Xianbiao Fu†,[1] Aoni Xu†,[1] Jakob B. Pedersen,[1] Shaofeng Li,[1] Rokas Sažinas,[1] Yuanyuan Zhou,[1] Suzanne Z. Andersen,[1] Mattia Saccoccio,[1] Niklas H. Deissler,[1] Jon Bjarke Valbæk Mygind,[1] Jakob Kibsgaard,[1] Peter C. K. Vesborg,[1] Jens K. Nørskov*,[1] Ib Chorkendorff*[1]

[1.] Department of Physics, Technical University of Denmark, Kongens Lyngby, Denmark

†These authors contributed equally to this work

*Corresponding author. Email: jkno@dtu.dk (J.K.N.), ibchork@fysik.dtu.dk (I.C.)

Table of Contents

Supplementary Figures 1-48

Supplementary Tables 1-2

Supplementary References 1-5

Supplementary Figures

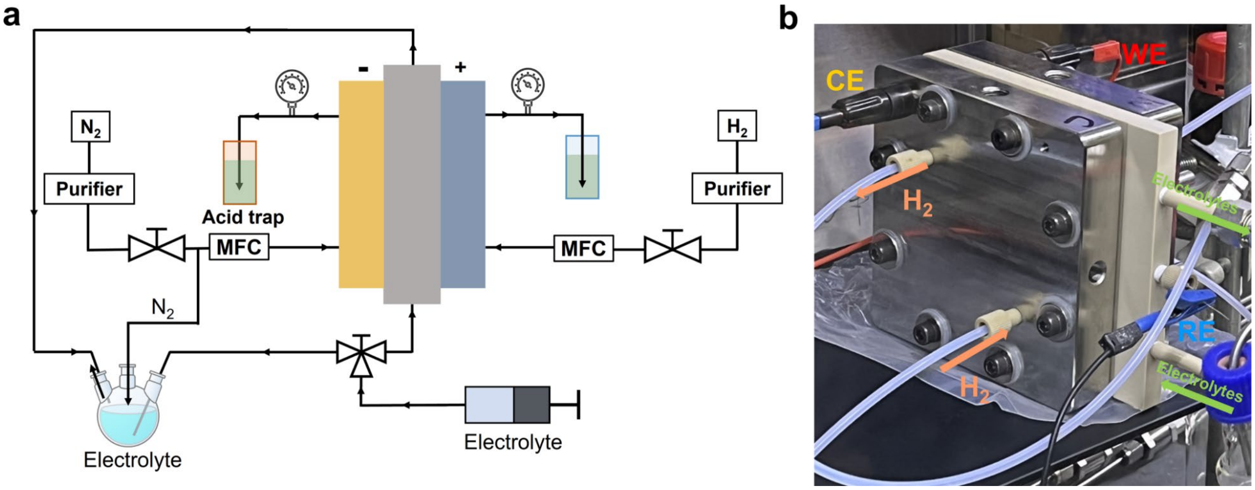

Supplementary Fig. 1. Schematic diagram (a) and photo (b) of the continuous-flow reactor setup. The pressure gradient of 15 mbar between the gas inlet and outlet of the flow cell was modulated by a 10.5 centimeters water column (50 ml) above the gas. The dimension of the flow cell was 10.7 cm×10.7 cm×5.1 cm. The effective area of the flow field was 25 cm^2. The gasket is then placed on top of the electrolytes chamber (PEEK), followed by an SSC and PtAu/SSC used as the working electrode and counter electrode, respectively. The cell is compressed by bolt tightening.

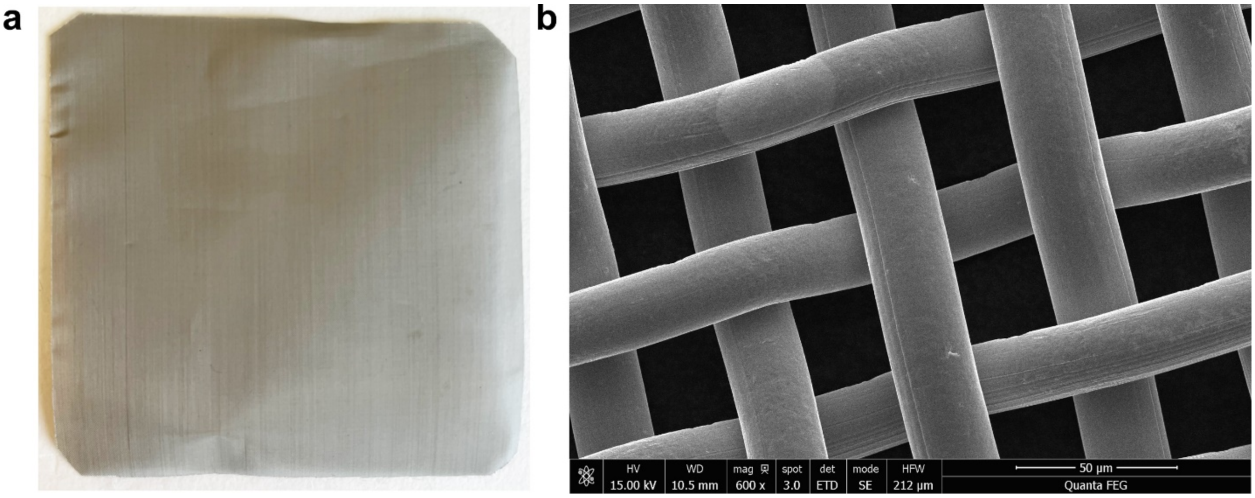

Supplementary Fig. 2. Photo (a) and SEM image (b) of 30 µm SSC.

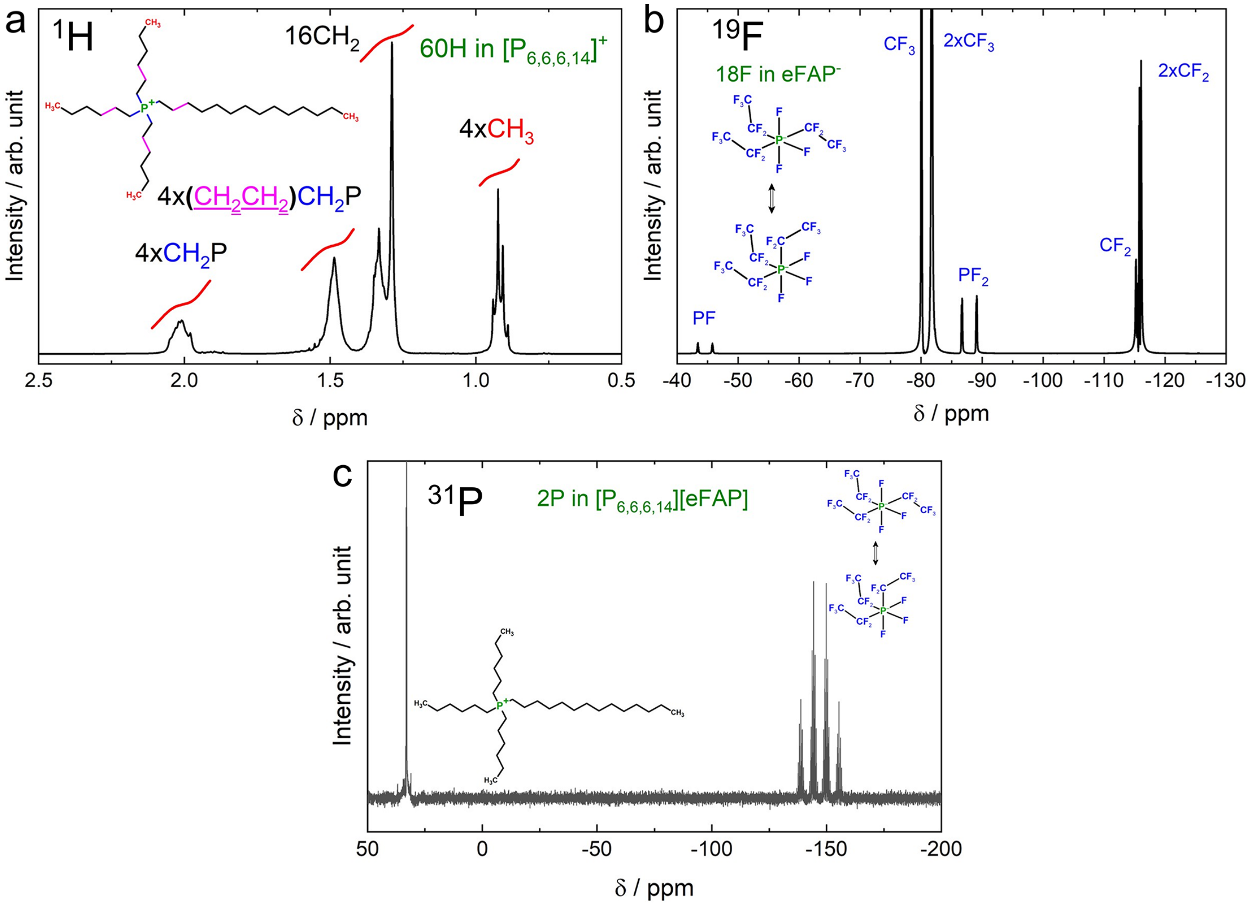

Supplementary Fig. 3. (a) ^{1}H NMR, (b) ^{19}F NMR, and (c) ^{31}P NMR data of $[P_{6,6,6,14}]$[eFAP]. NMR results confirmed that we successfully prepared the phosphonium-based salts ($[P_{6,6,6,14}]$[eFAP]) and no starting material or contamination.

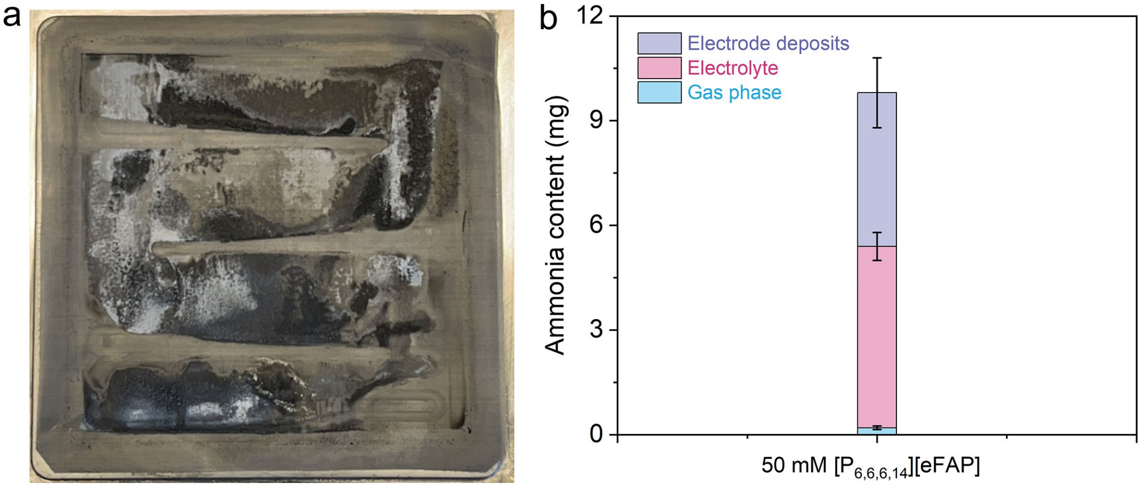

Supplementary Fig. 4. (a) Picture of the working electrode after the CP test of 700 C charge using phosphonium salts [$P_{6,6,6,14}$][eFAP]. (b) The distribution of produced ammonia in the electrolyte, gas phase, and electrode deposits. After passing a charge of 700 C (over 2.5 h), about 40% of the produced ammonia was trapped in the electrode deposits, which means the proton shuttle cannot protonate LiN_xH_y in time and the LiN_xH_y is accumulated. Therefore, the lower performance of phosphonium cation is attributed to the difficulty of carrying protons and lower diffusion rate. Error bars represent the mean ± standard deviation derived from three independent measurements.

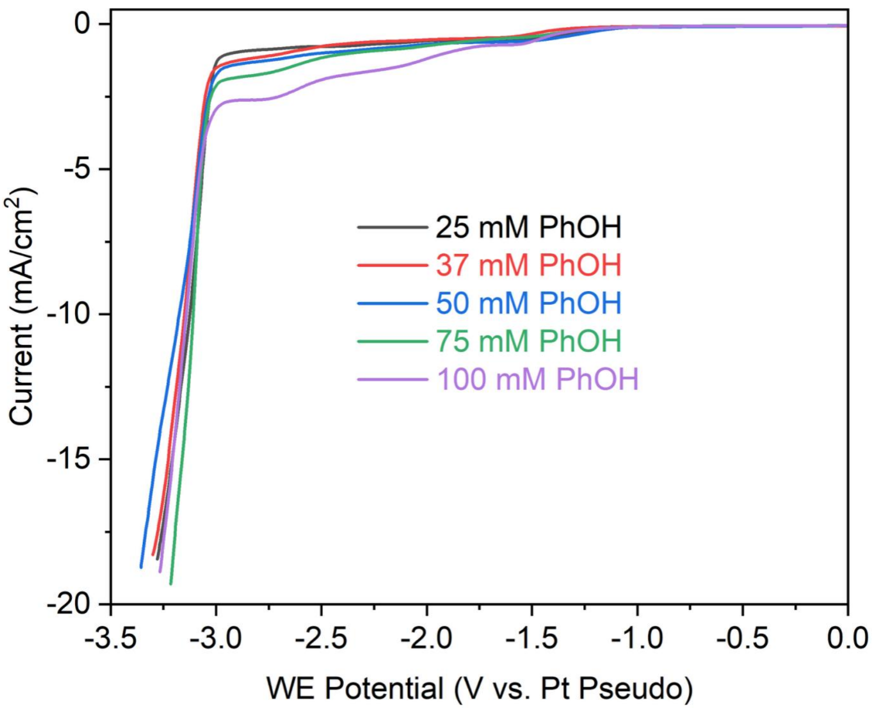

Supplementary Fig. 5. LSV curves were obtained at different phenol concentrations with 80% *iR* correction. The working electrode is the 30 μm SSC. The electrolyte is 1 M $LiBF_4$ in THF with different phenol concentrations. Different phenol concentrations can change the available proton concentration in the electrolyte due to the presence of protons within PhOH. The higher the concentration of PhOH can lead to the higher the current density of HER.

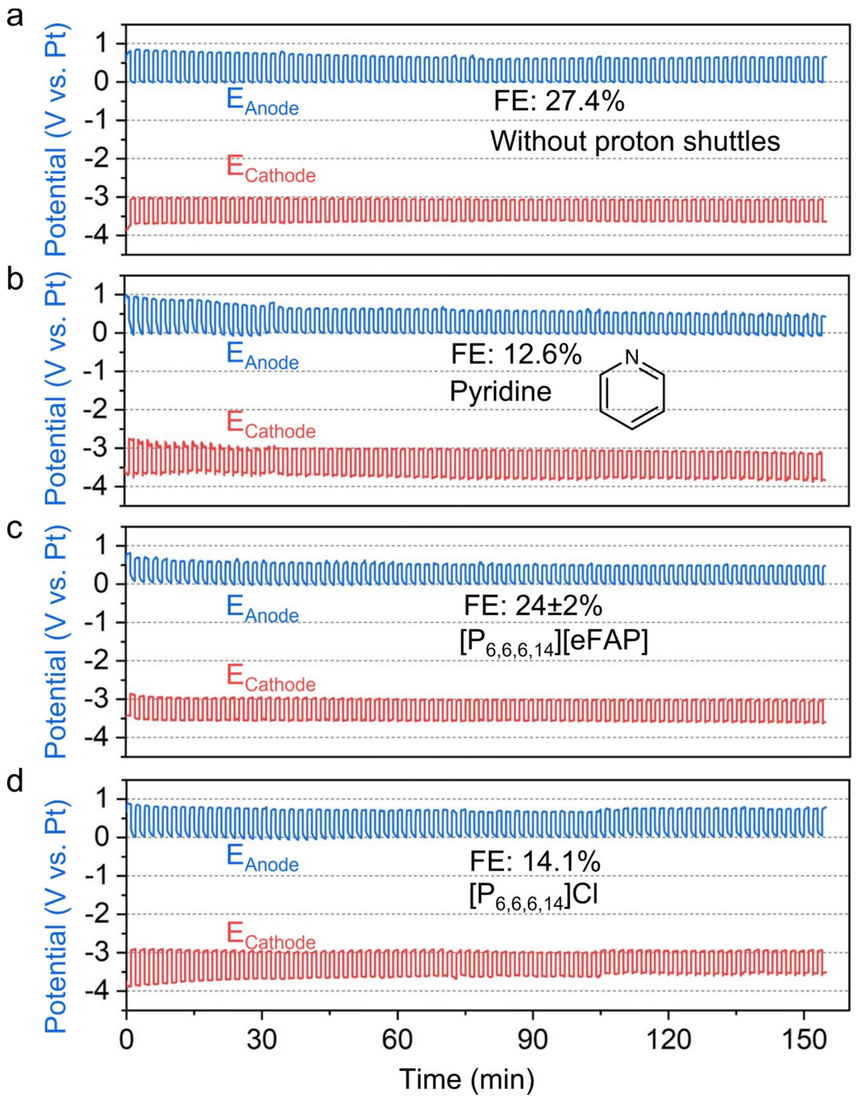

Supplementary Fig. 6. CP curves using different proton shuttles. (a) Without proton shuttle. (b) Pyridine. (c) $[P_{6,6,6,14}]$[eFAP]. (d) $[P_{6,6,6,14}]$Cl. The red and blue lines represent the WE (cathode) potential and CE (anode) potential, respectively. All potentials are without *iR* correction. The electrolyte is 1 M $LiBF_4$ in THF with different proton shuttles. The potential cycling condition setting is 1 minute for Li deposition and 1 minute for resting at OCV. The resting potential in the absence of the proton shuttle was -3 V versus Pt, which can be the benchmark for resting potential when the presence of the proton shuttle. The data represent the mean ± standard deviation derived from three independent measurements.

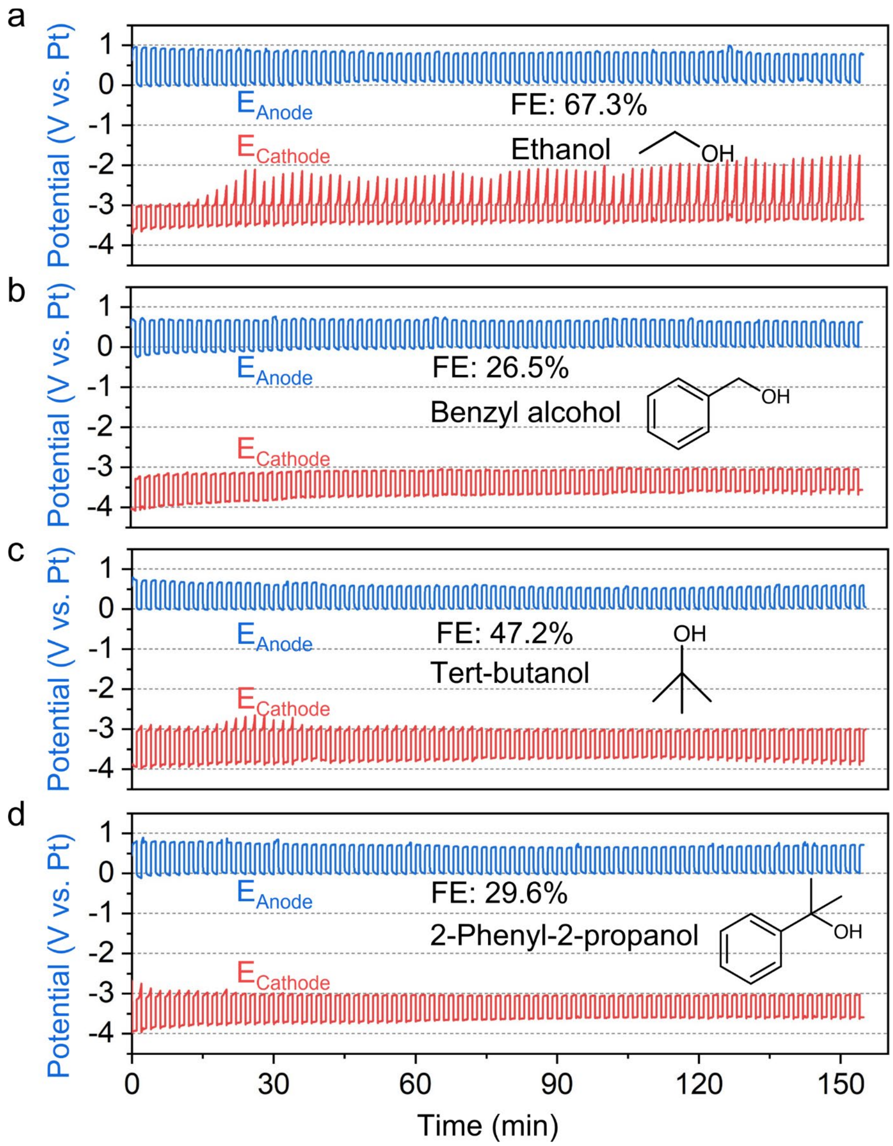

Supplementary Fig. 7. CP curves using different proton shuttles. (a) Ethanol (EtOH). (b) Benzyl alcohol. (c) Tert-butanol. (d) 2-phenyl-2-propanol. The red and blue lines represent the WE (cathode) potential and CE (anode) potential, respectively. All potentials are without *iR* correction. The electrolyte is 1 M $LiBF_4$ in THF with different proton shuttles. The potential cycling condition setting is 1 minute for Li deposition and 1 minute for resting at OCV. The resting potential of ethanol is -2 V versus Pt. The data represent the mean ± standard deviation derived from three independent measurements.

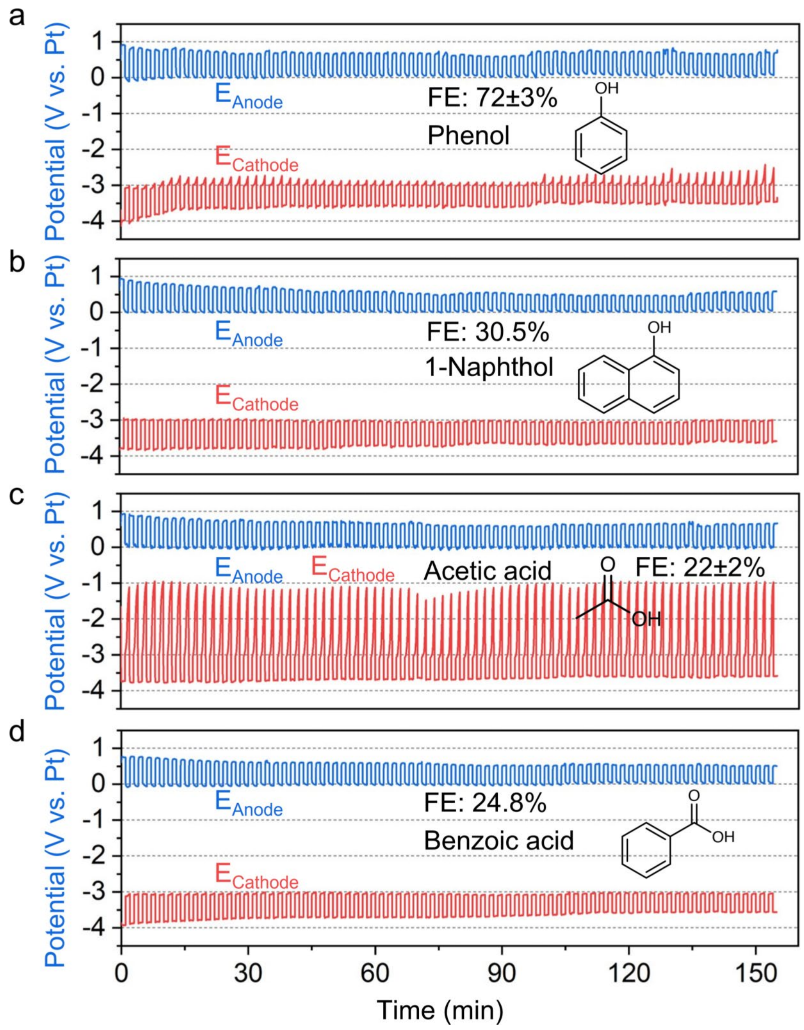

Supplementary Fig. 8. CP curves using different proton shuttles. (a) Phenol (PhOH). (b) 1-Naphthol. (c) Acetate acid. (d) Benzoic acid. The red and blue lines represent the WE (cathode) potential and CE (anode) potential, respectively. All potentials are without *iR* correction. The electrolyte is 1 M $LiBF_4$ in THF with different proton shuttles. The potential cycling condition setting is 1 minute for Li deposition and 1 minute for resting at OCV. The resting potential of acetate acid is -1 V versus Pt. The data represent the mean ± standard deviation derived from three independent measurements.

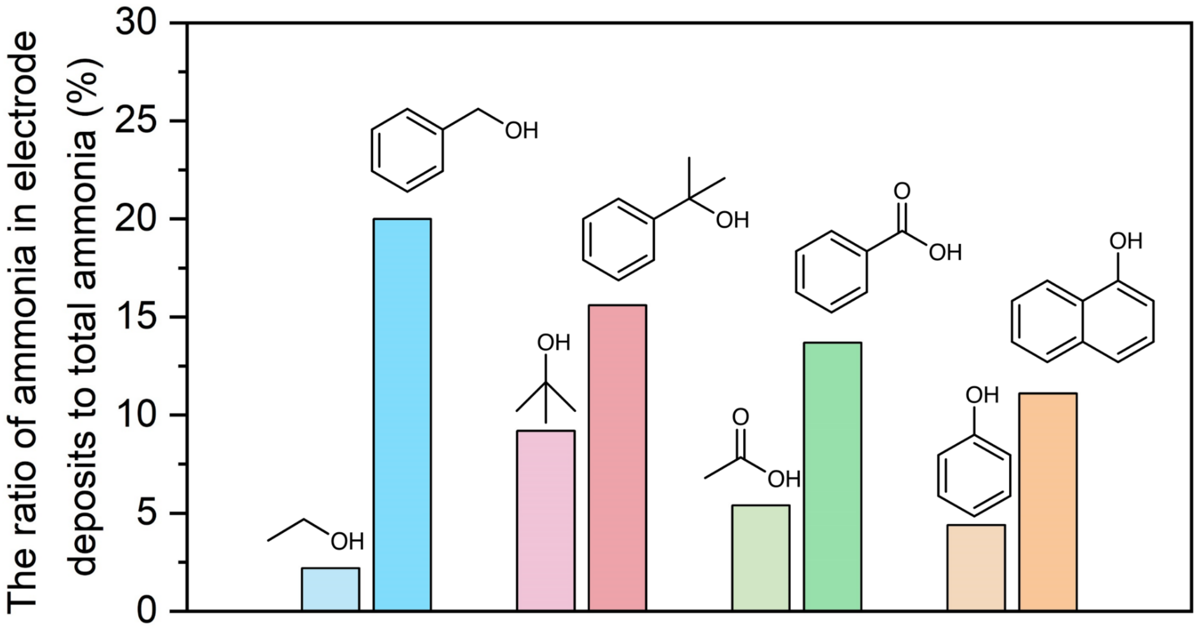

Supplementary Fig. 9. The ratio of ammonia in electrode deposits to total ammonia. The higher ratio of ammonia in electrode deposits to total ammonia (i.e., proton shuttle cannot protonate LiN_xH_y in time and accumulated the LiN_xH_y) is observed when proton shuttles are substituted by larger groups. This higher ratio could be indicative of a lower protonation ability of the proton shuttle.

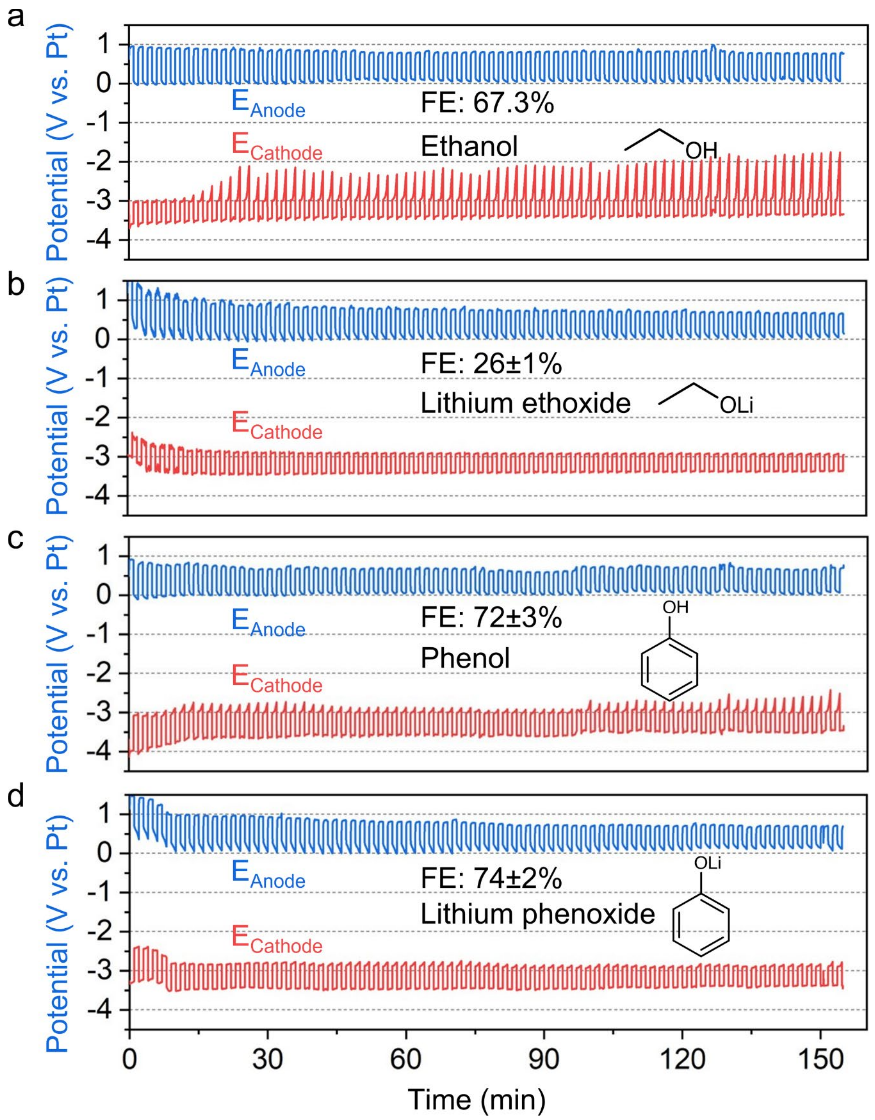

Supplementary Fig. 10. CP curves using different proton shuttles. (a) Ethanol (EtOH). (b) Lithium ethoxide (EtOLi). (c) Phenol (PhOH). (d) Lithium phenoxide (PhOLi). The red and blue lines represent the WE (cathode) potential and CE (anode) potential, respectively. All potentials are without *iR* correction. The electrolyte is 1 M $LiBF_4$ in THF with different proton shuttles. The potential cycling condition setting is 1 minute for Li deposition and 1 minute for resting at OCV. The data represent the mean ± standard deviation derived from three independent measurements.

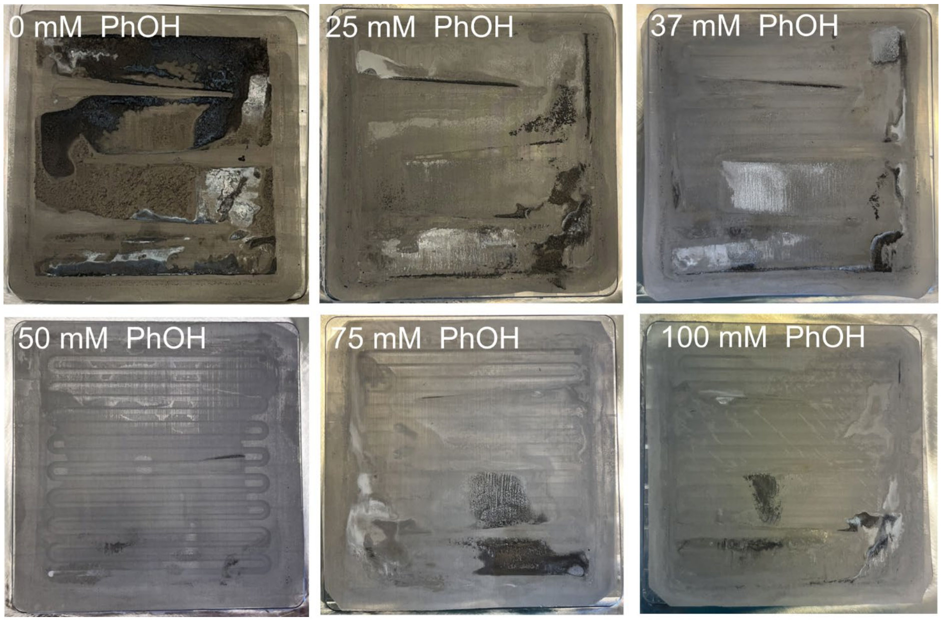

Supplementary Fig. 11. Pictures of the working electrode after the CP test of 700 C charge at different PhOH concentrations. The electrolyte is 1 M $LiBF_4$ in THF with different PhOH concentrations. The potential cycling condition setting is 1 minute for Li deposition and 1 minute for resting at OCV. Without PhOH in the electrolyte, a thick deposit (metallic Li) was observed on the front side facing the electrolyte due to the proton limitation (lack of the proton shuttle). In the presence of PhOH in the electrolyte, the visible deposits are greatly decreased.

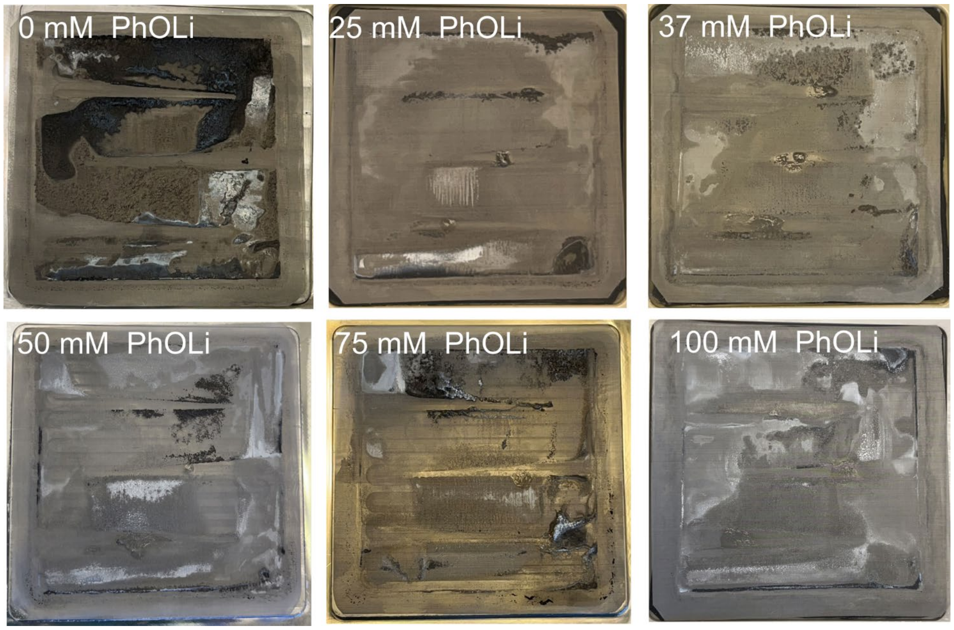

Supplementary Fig. 12. Pictures of the working electrode after the CP test of 700 C charge at different PhOLi concentrations. The electrolyte is 1 M $LiBF_4$ in THF with different PhOLi concentrations. The potential cycling condition setting is 1 minute for Li deposition and 1 minute for resting at OCV. Without PhOLi in the electrolyte, a thick deposit (metallic Li) was observed on the front side facing the electrolyte due to the proton limitation (lack of the proton shuttle). In the presence of PhOLi in the electrolyte, the visible deposits are greatly decreased.

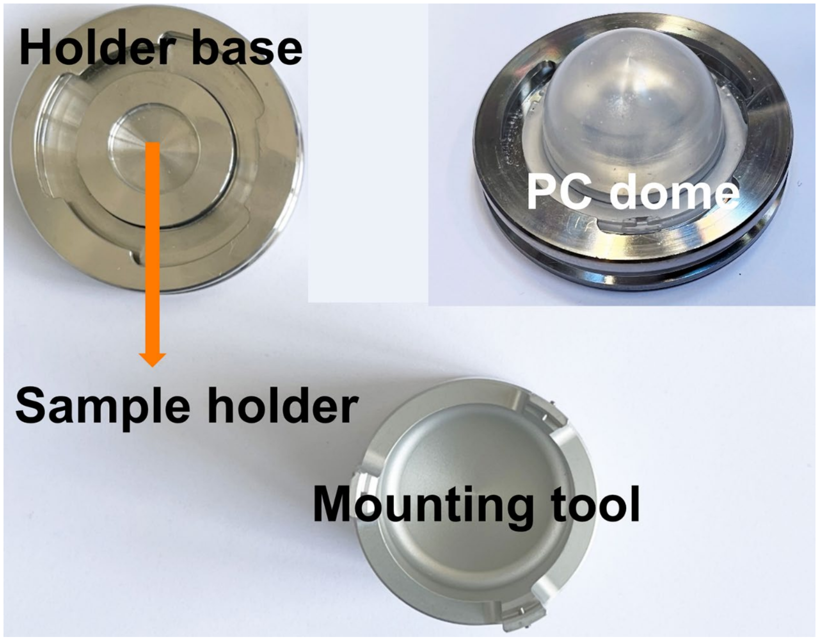

Supplementary Fig. 13. Pictures of XRD domed sample holder with polycarbonate (PC) dome without exposure to air. The thin and x-ray transparent PC dome is tightened onto the holder base (Anton Paar, Cat. No. 132598), and the airtightness is ensured by an O-ring between the dome and sample holder.

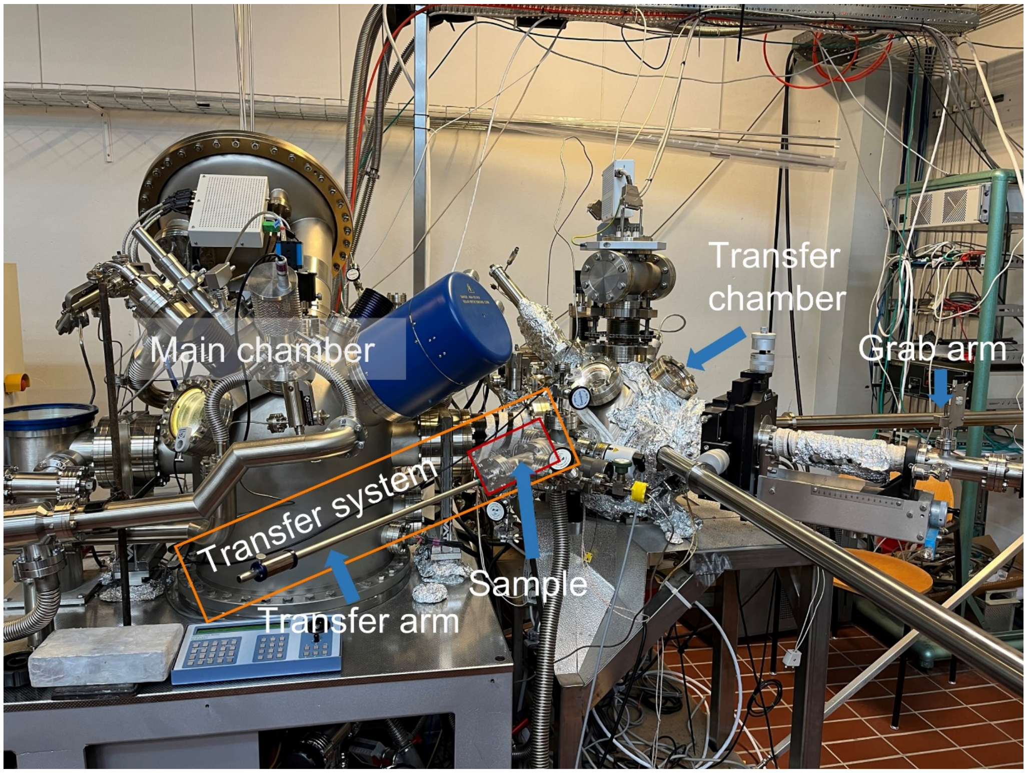

Supplementary Fig. 14. Home-built XPS transfer system. The transfer system was first loaded into an Ar glovebox for sample loading, and the gate valve on the system was closed. Then the system was attached to the transfer chamber and pumped down. When the pressure of the transfer system reached below 5×10^{-6} mbar, the transfer gate was opened, and the sample was introduced to the transfer chamber. Finally, the grab arm was used to catch the sample and transfer it to the analysis chamber.

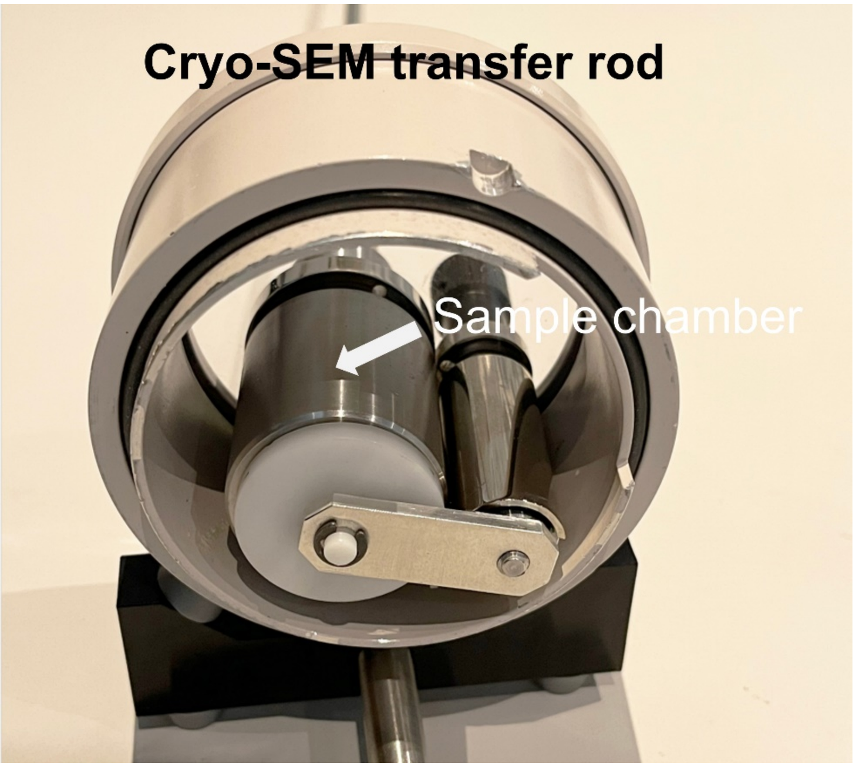

Supplementary Fig. 15. Picture of the Cryo-SEM transfer rod. The transfer rod was first loaded into an Ar glovebox for sample loading, and the gate valve on the system was closed. The cryo-SEM system was used to transfer the samples to the microscope chamber for SEM imaging.

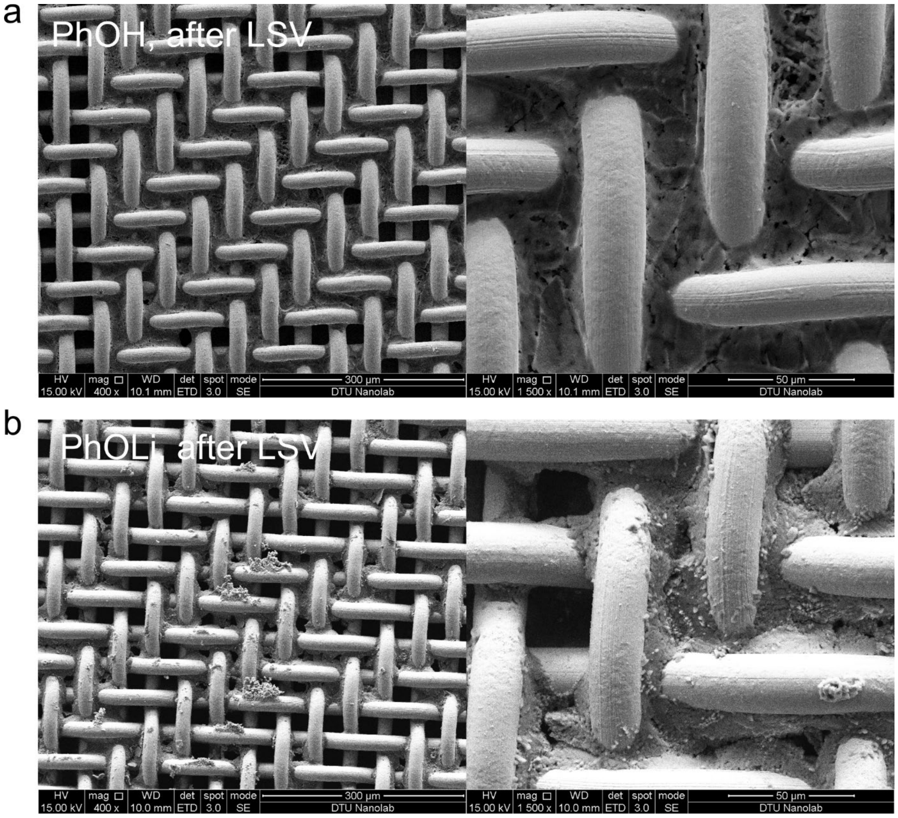

Supplementary Fig. 16. SEM images of working electrodes (cathode) after LSV. (a) SEM image of SEI layer after LSV with PhOH (37 mM). (b) SEM image of SEI layer after LSV with PhOLi (37 mM). The SEM images show a uniform SEI layer formed after the LSV test with PhOH, while fewer deposition species with PhOLi.

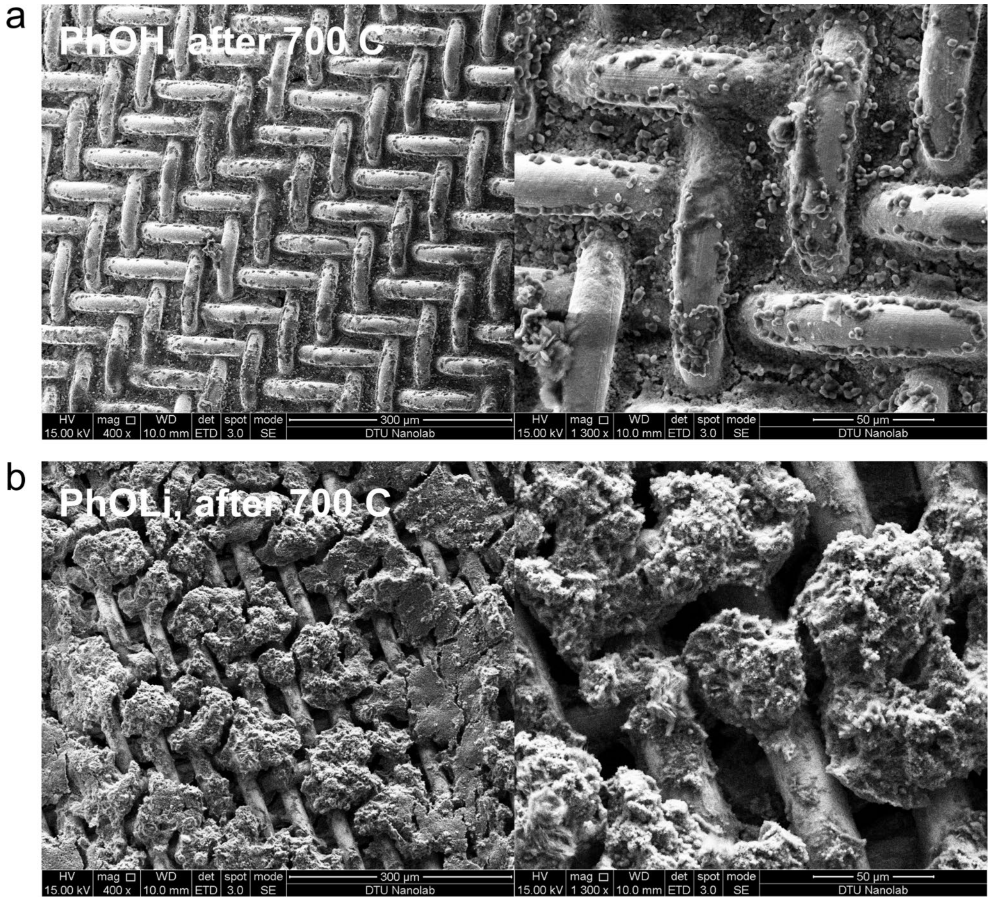

Supplementary Fig. 17. SEM images of working electrodes (cathode) after CP test of 700 C. (a) SEM image of post-reaction electrode deposits (after 700 C) with PhOH (37 mM). (b) SEM image of post-reaction electrode deposits (after 700 C) with PhOLi (37 mM). After a total charge of 700 C in the CP test, the cathode surface of PhOLi exhibited an irregular and thicker layer of deposits, while a uniform and thinner layer of deposits was observed when using PhOH.

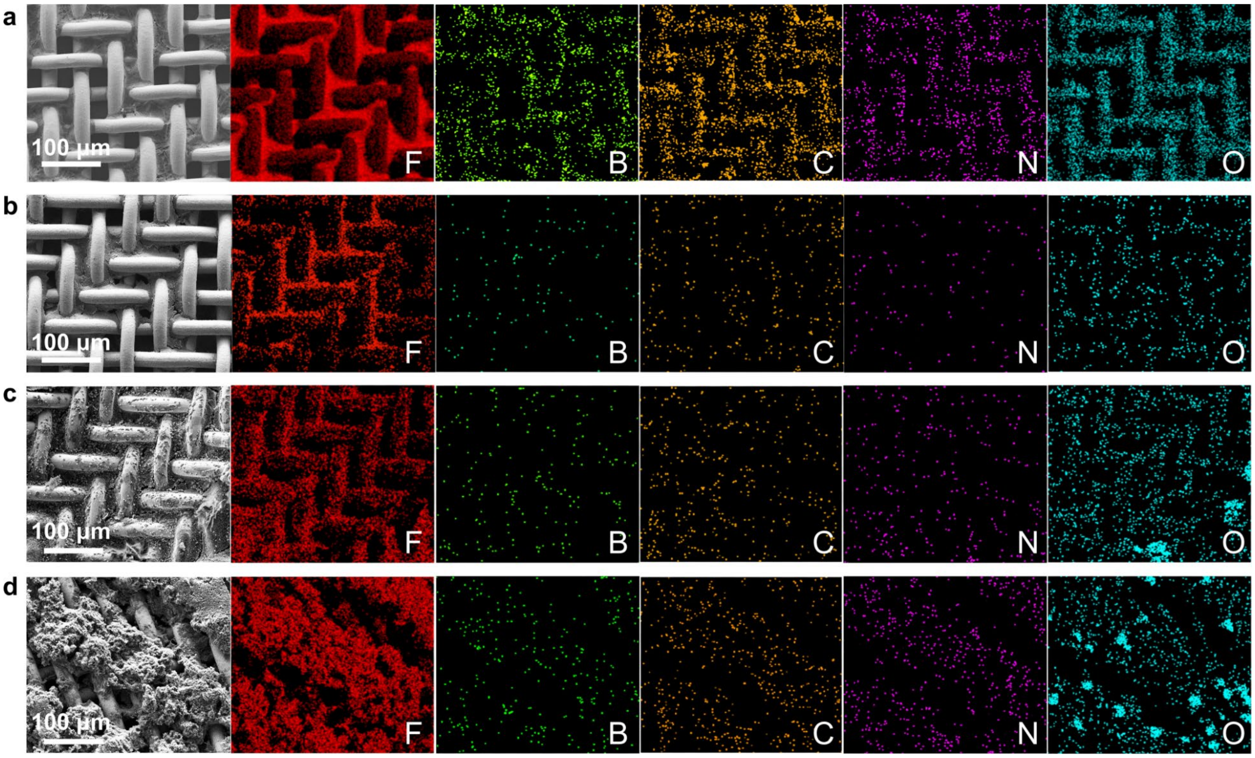

Supplementary Fig. 18. SEM-EDS mapping of post-reaction electrode deposits and SEI layer. (a) After LSV with PhOH (37 mM). (b) After LSV with PhOLi (37 mM). (c) After the CP test of 700 C with PhOH (37 mM). (d) After the CP test of 700 C with PhOLi (37 mM). The high EDS signals of F suggest more fluoride on the working electrodes.

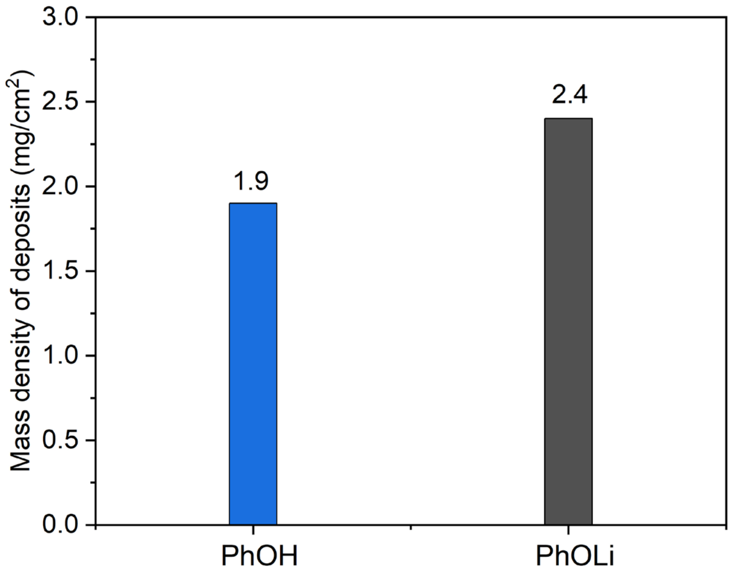

Supplementary Fig. 19. Mass density of cathode deposits for PhOH (37 mM) and PhOLi (37 mM) after the CP test of 700 C.

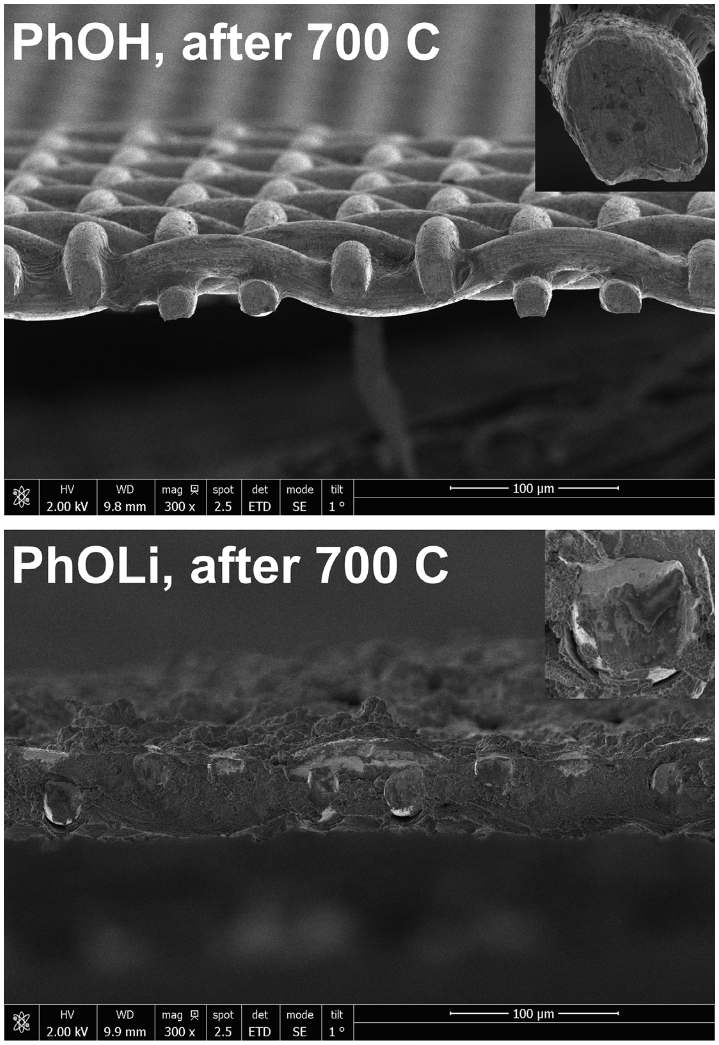

Supplementary Fig. 20. SEM images of post-reaction electrode deposits for PhOH (37 mM) and PhOLi (37 mM) after the electrochemical test.

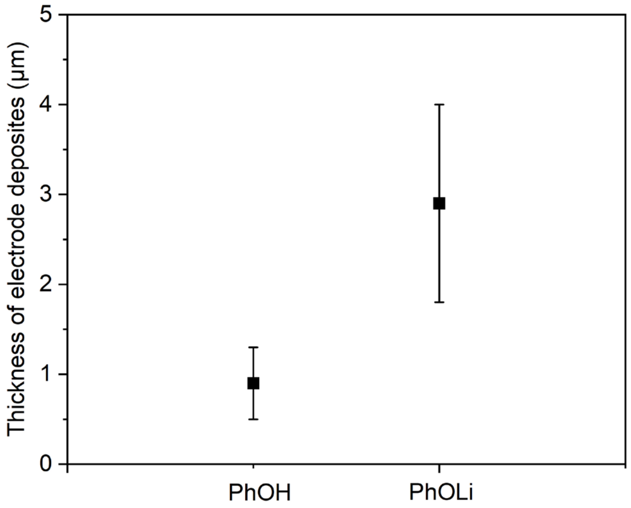

Supplementary Fig. 21. The thickness of the cathode deposits layer on the stainless-steel wires when using PhOH (37 mM) or PhOLi (37 mM) after the CP test of 700 C, respectively. Error bars represent the mean ± standard deviation derived from 10 independent measurements.

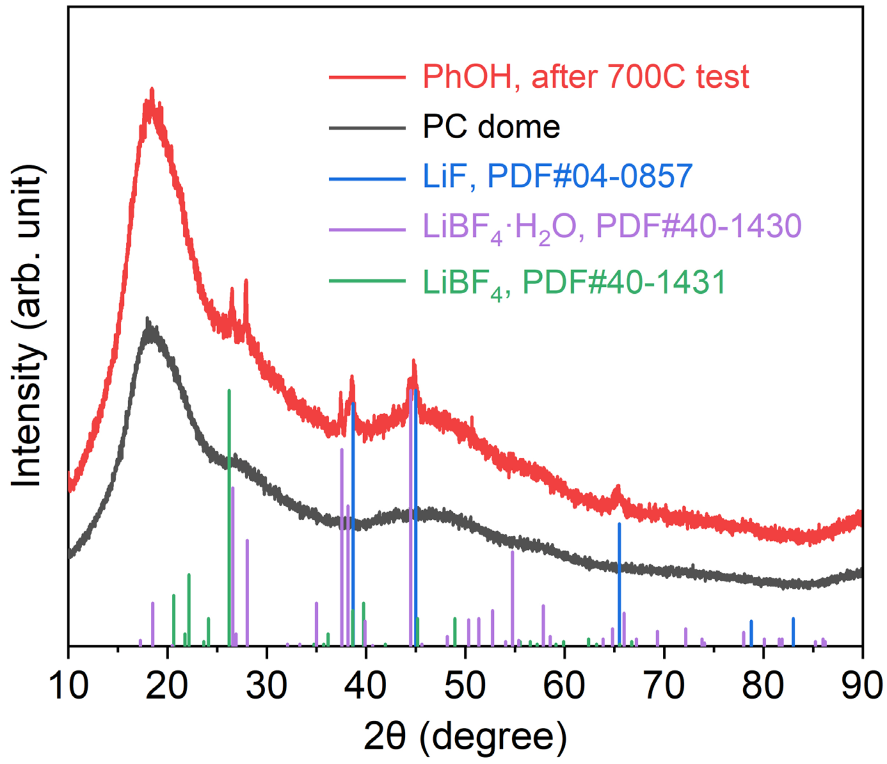

Supplementary Fig. 22. XRD pattern of deposit collected from post-reaction electrode deposits after the CP test of 700 C with PhOH (37 mM). The XRD pattern confirms the main component is lithium fluoride (LiF).

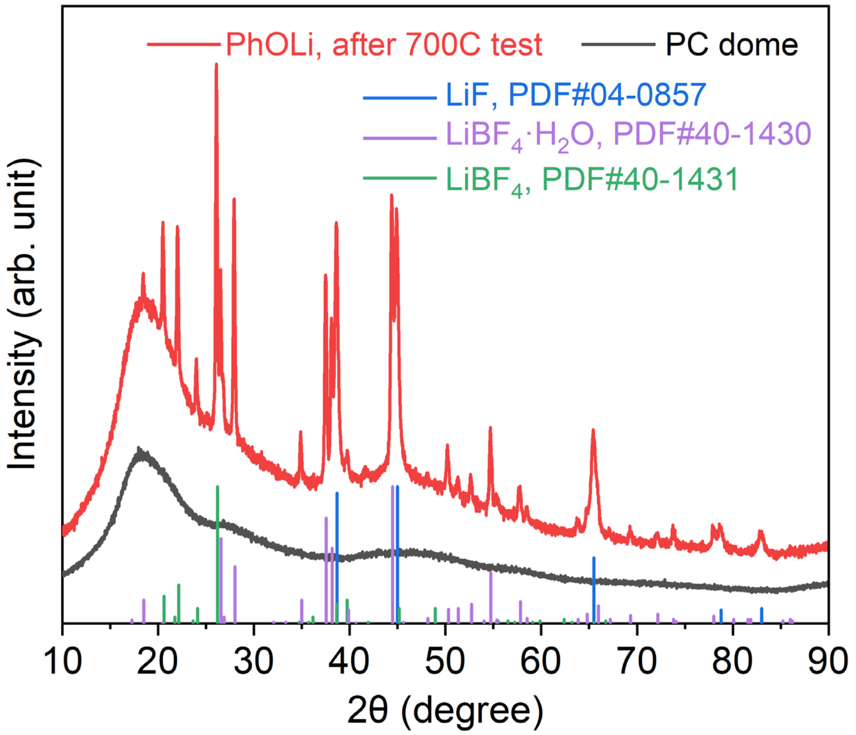

Supplementary Fig. 23. XRD pattern of deposit collected from post-reaction electrode deposits after the CP test of 700 C with PhOLi (37 mM). The XRD pattern confirms the main component is lithium fluoride (LiF).

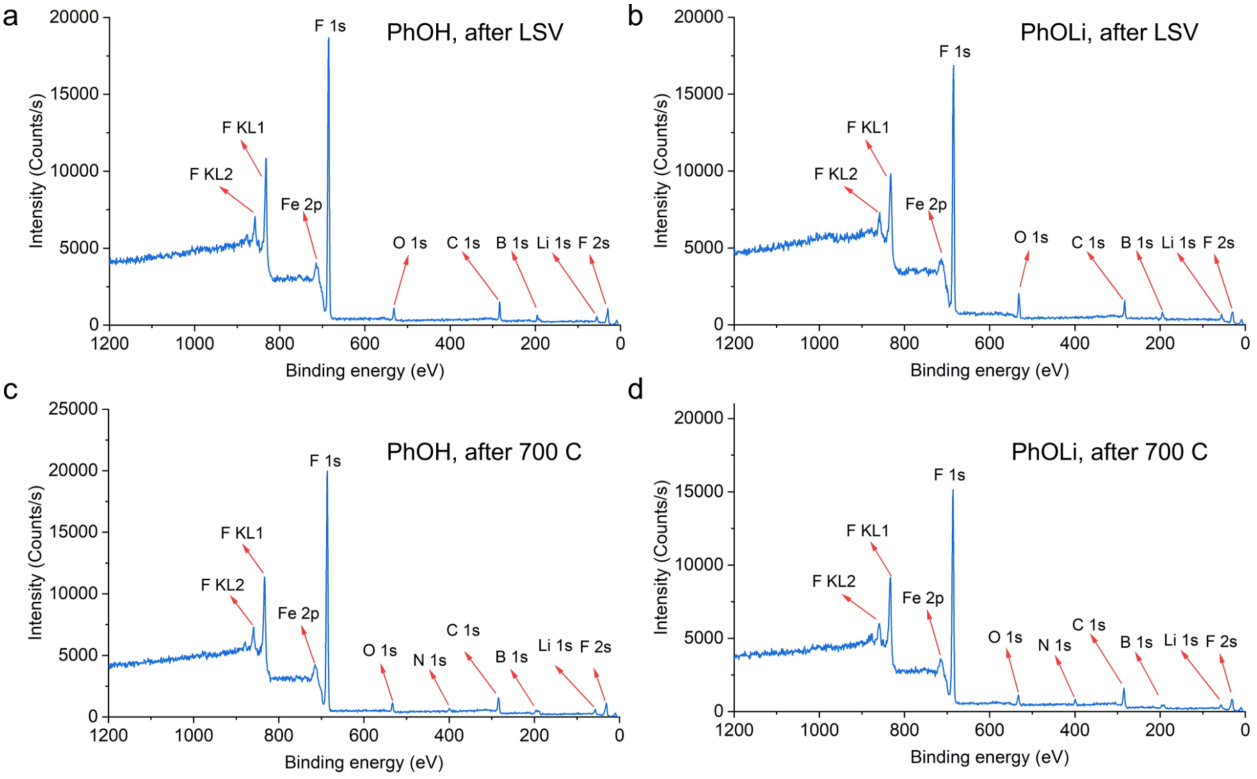

Supplementary Fig. 24. XPS survey spectra of the SEI layer after LSV and post-reaction electrode deposits after the CP test of 700 C. (a) After LSV with PhOH (37 mM). (b) After LSV with PhOLi (37 mM). (c) After the CP test of 700 C with PhOH (37 mM). (d) After the CP test of 700 C with PhOLi (37 mM).

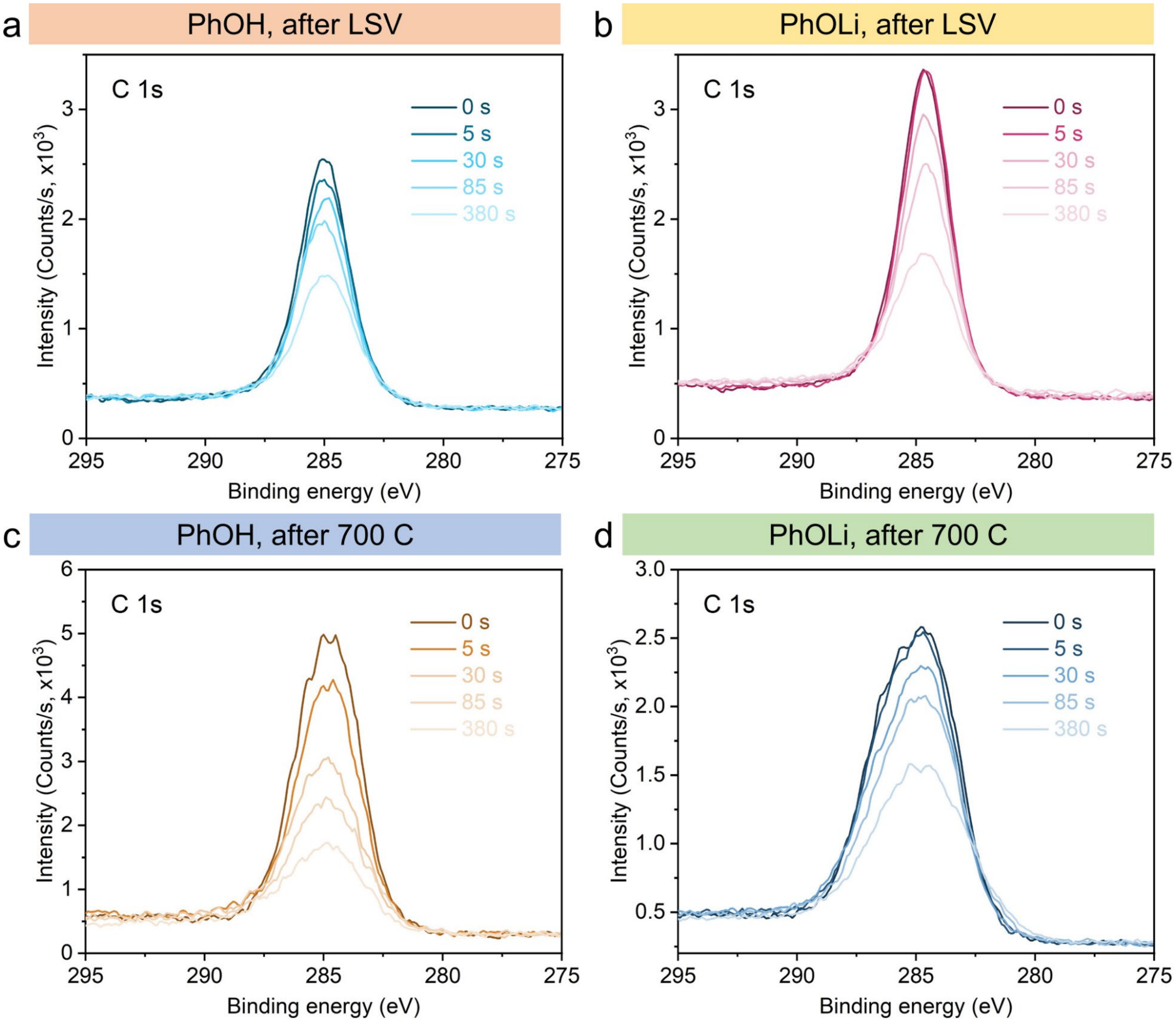

Supplementary Fig. 25. Depth-profiling XPS spectra of C 1s at different argon sputtering times. (a) After LSV with PhOH (37 mM). (b) After LSV with PhOLi (37 mM). (c) After the CP test of 700 C with PhOH (37 mM). (d) After the CP test of 700 C with PhOLi (37 mM).

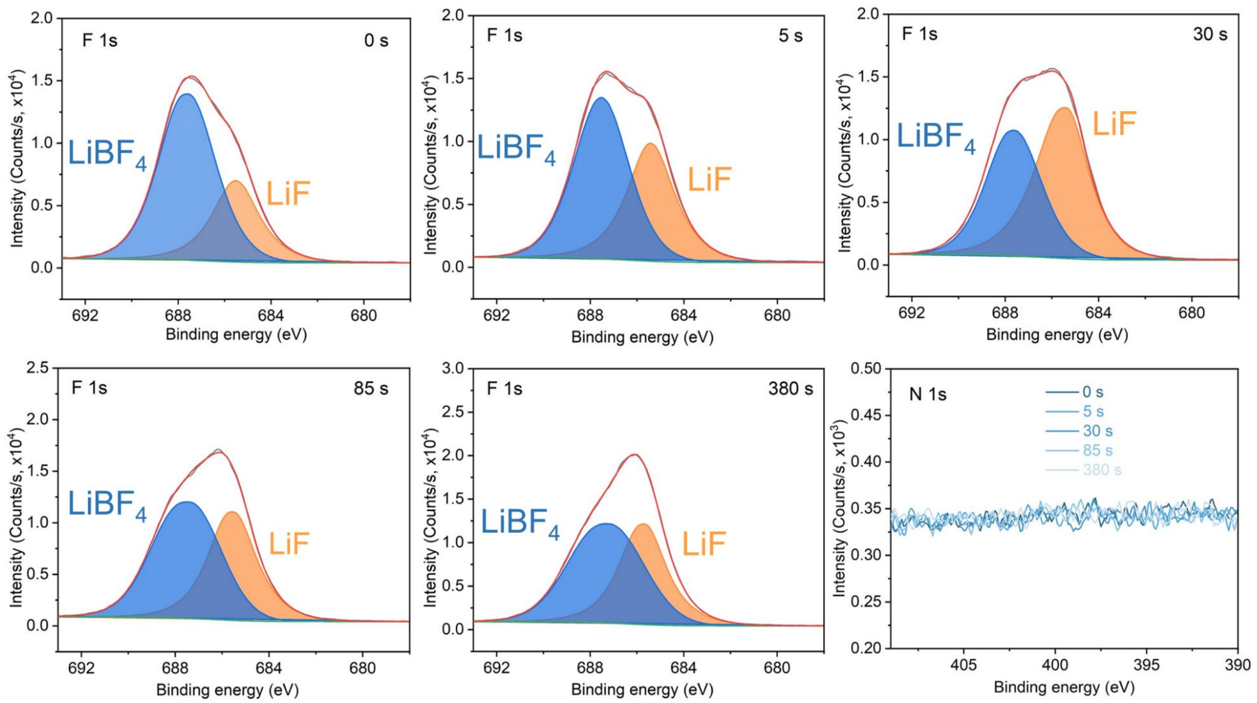

Supplementary Fig. 26. Depth-profiling XPS spectra of F 1s for SEI layer after LSV with PhOH (37 mM) at different argon sputtering times.

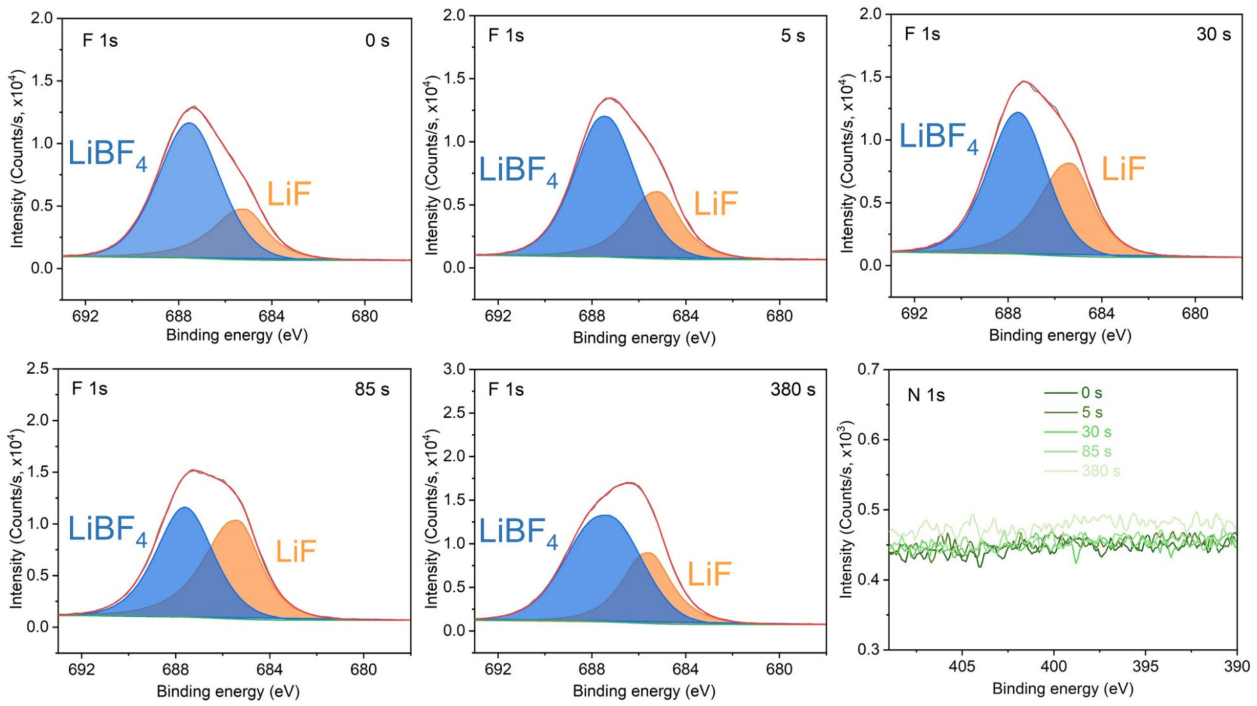

Supplementary Fig. 27. Depth-profiling XPS spectra of F 1s for SEI layer after LSV with PhOLi (37 mM) at different argon sputtering times.

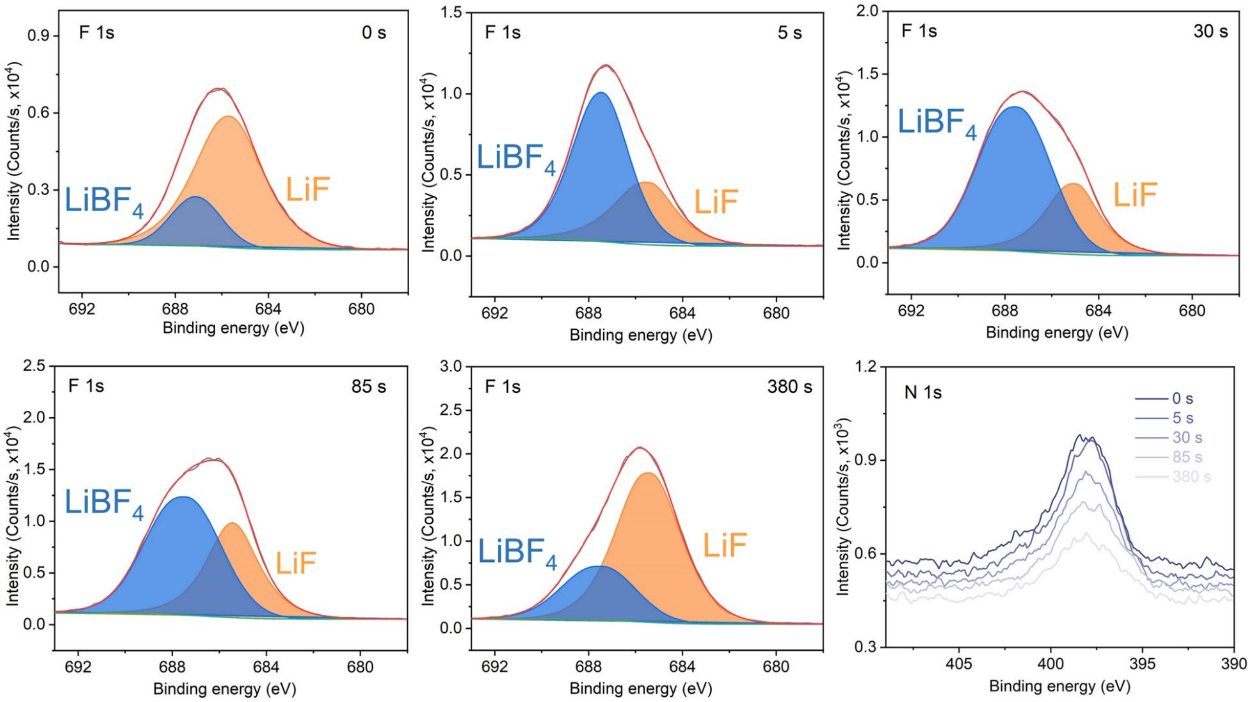

Supplementary Fig. 28. Depth-profiling XPS spectra of F 1s for the post-reaction electrode deposits after the CP test of 700 C with PhOH (37 mM) at different argon sputtering times.

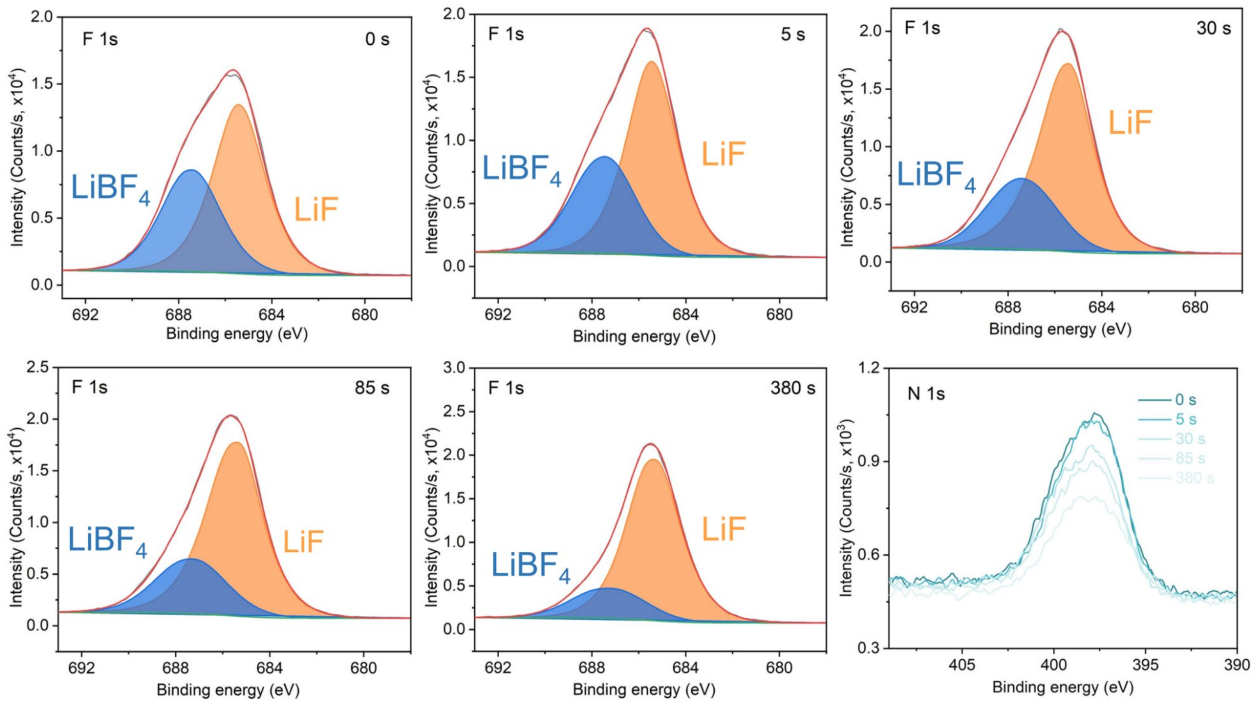

Supplementary Fig. 29. Depth-profiling XPS spectra of F 1s for the post-reaction electrode deposits after the CP test of 700 C with PhOLi (37 mM) at different argon sputtering times.

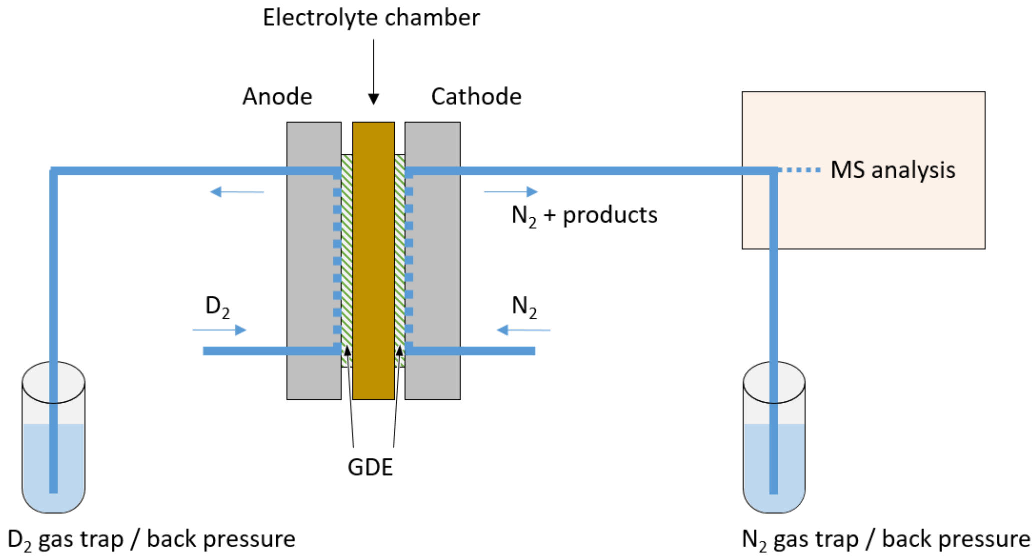

Supplementary Fig. 30. Schematic of flow cell measurements in connection with mass spectrometry. D_2 is passed over the anode gas diffusion electrode (GDE) and flows into a back pressure vessel. On the cathode, N_2 is flowing over the GDE and led into the mass spectrometer (MS) for product detection. Outlet gas is then passed into a backpressure vessel.

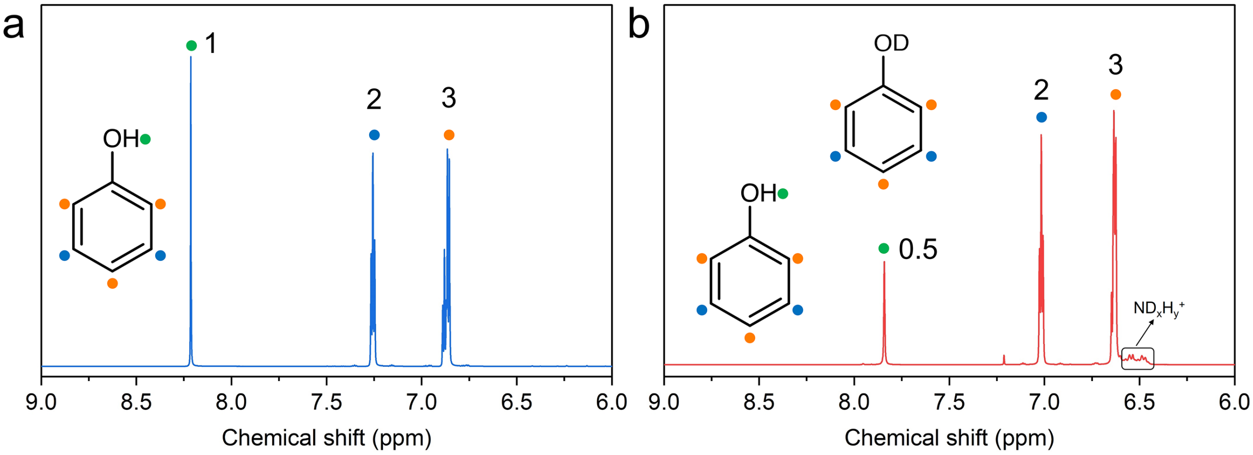

Supplementary Fig. 31. The ^{1}H NMR spectra of deuterium isotope-labeled experiment. (a) The ^{1}H NMR spectra of PhOH in tetrahydrofuran before the electrochemistry test. The peak area ratio is 1:2:3. (b) The ^{1}H NMR spectra of post-reaction electrolytes using PhOH as a proton shuttle and feeding D_2 to the anode side. The peak area ratio is 0.5:2:3.

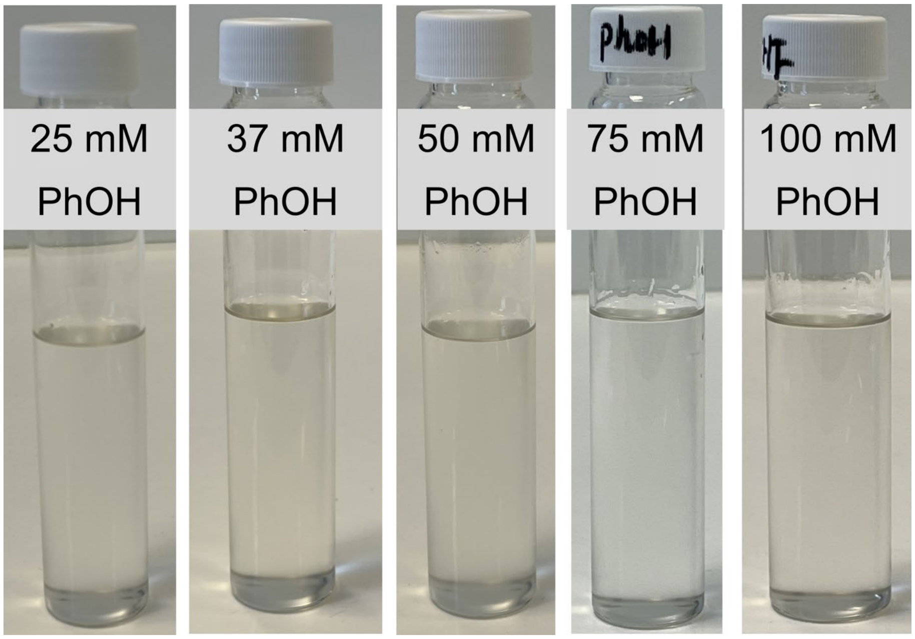

Supplementary Fig. 32. Pictures of the electrolytes after the CP test of 700 C charge at different PhOH concentrations.

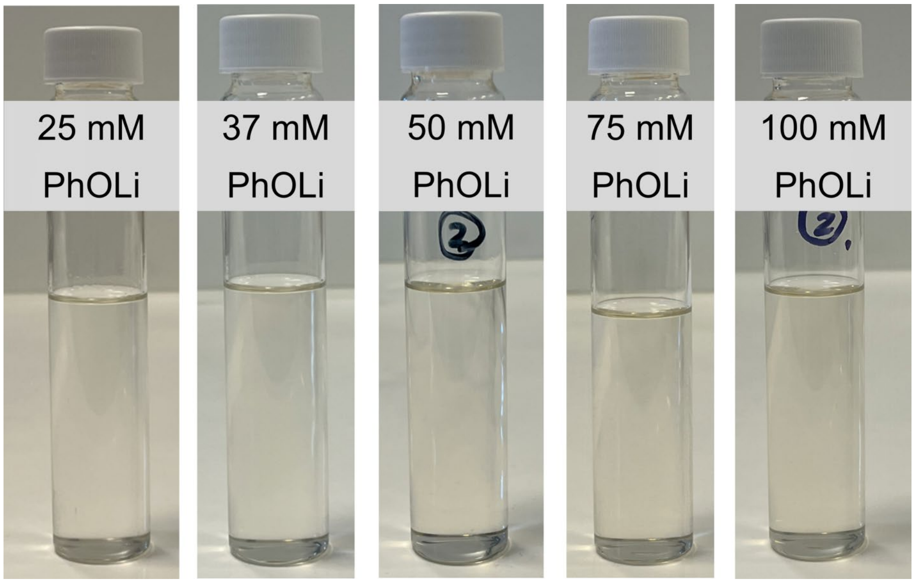

Supplementary Fig. 33. Pictures of the electrolytes after the CP test of 700 C charge at different PhOLi concentrations.

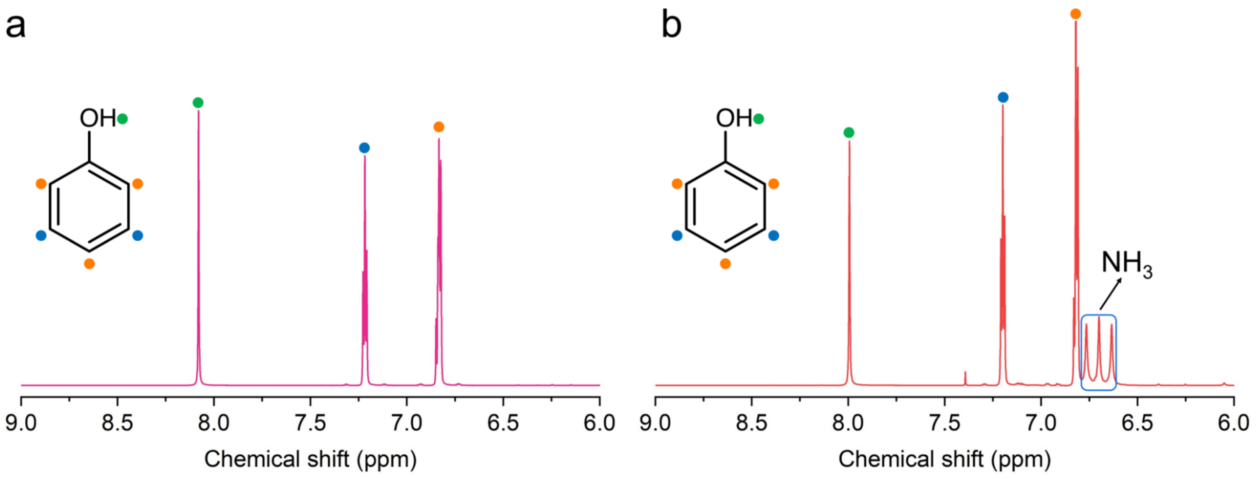

Supplementary Fig. 34. The ^{1}H NMR spectra of electrolytes before and after the CP test. (a) ^{1}H NMR spectra of electrolytes with PhOH before the CP test. (b) ^{1}H NMR spectra of electrolytes with PhOH after the CP test.

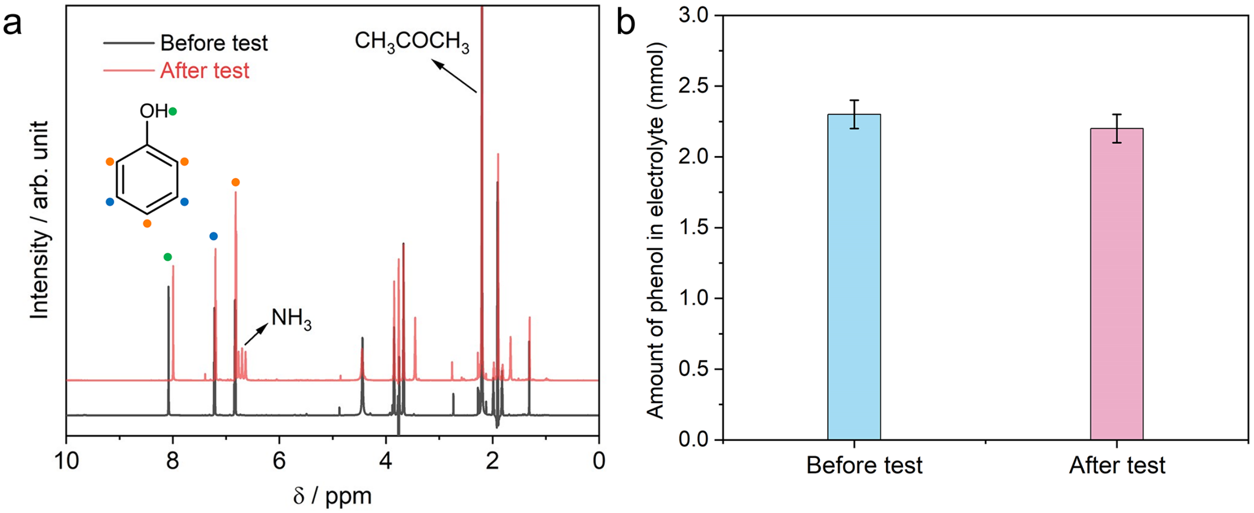

Supplementary Fig. 35. Quantifying the amount of PhOH before and after the CP test. (a) ^{1}H NMR spectra of electrolytes with PhOH before and after the CP test. (b) Quantifying the amount of PhOH before and after the CP test. For quantitative analysis, acetone (known concentration) was added to the electrolytes as a calibration compound. Quantitative NMR measurements indicate that the amount of PhOH remains almost unchanged after the CP test, which confirms the stability of PhOH. After the electrochemical test, the new peaks at 1.6 ppm and 3.4 ppm are from the polytetrahydrofuran (polyTHF), which is a constant decomposition of the electrolyte.[1] Under similar conditions, about 10% of ethanol was consumed during the Li-NRR process.[1] The standard deviation shown among the results as error bars are generated from the integration of the peaks, the number of peaks chosen to integrate, and the number of nuclei chosen.

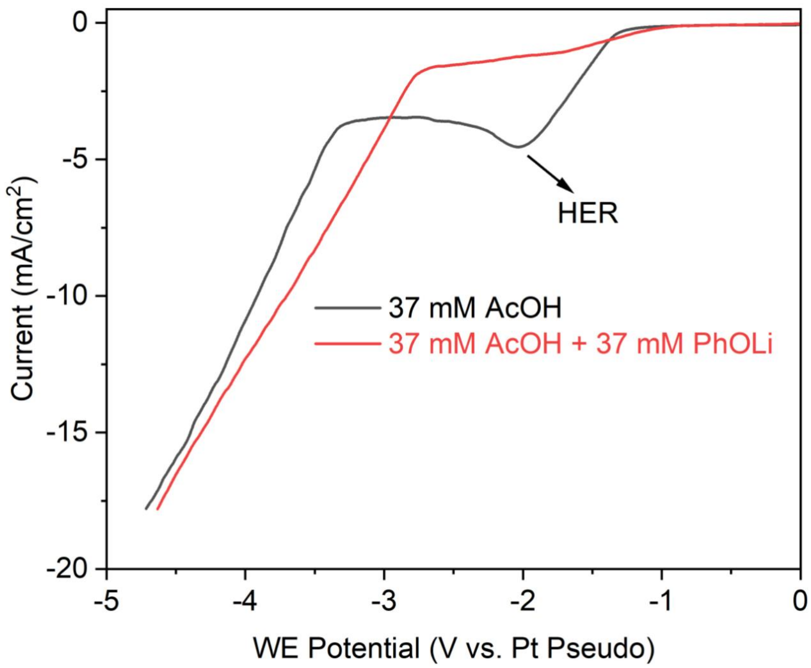

Supplementary Fig. 36. LSV curves were obtained with acetate acid (AcOH) or acetate acid mixed with PhOLi as proton shuttle without *iR* correction. The electrolyte is 1 M $LiBF_4$ in THF with acetate acid (AcOH) or a mixture of acetate acid with PhOLi. The proton-buffering capability of PhOLi (i.e., lowers the available proton concentration in the electrolyte) can effectively mitigate the adverse effects of the competitive HER.

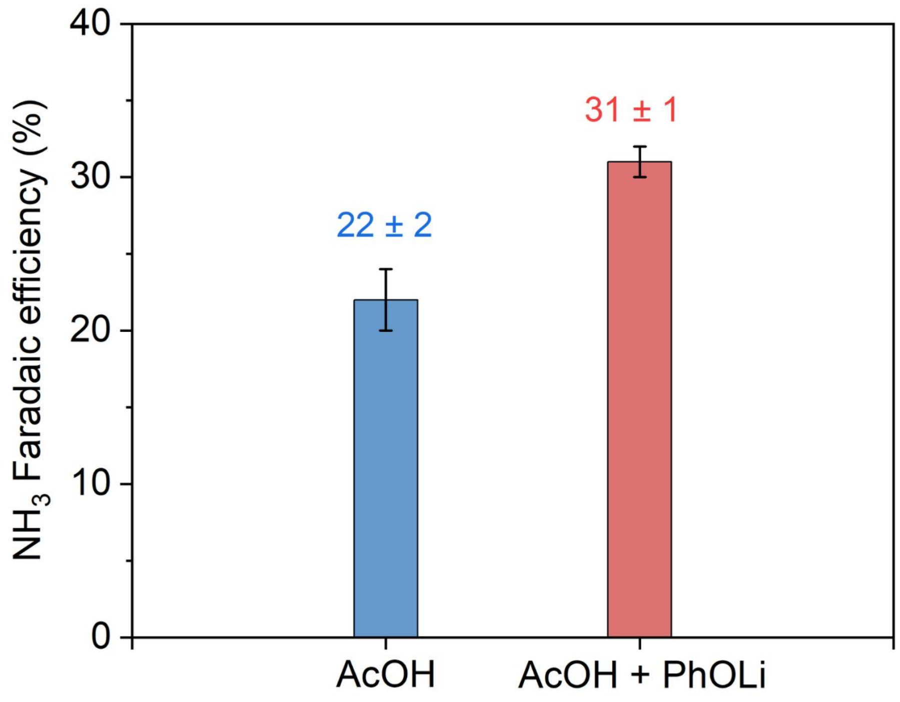

Supplementary Fig. 37. The comparison of FE between using acetate acid (AcOH) and acetate acid mixed with PhOLi as proton shuttle under similar experimental conditions. Error bars represent the mean ± standard deviation derived from three independent measurements.

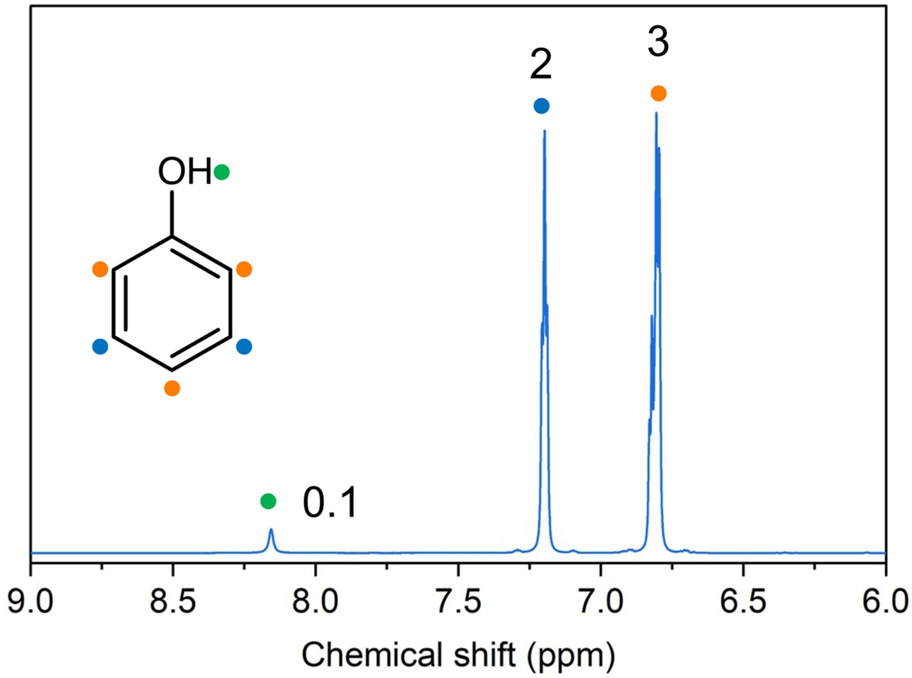

Supplementary Fig. 38. The ^{1}H NMR spectra of an equivalent mixture of acetate acid with PhOLi. The peak area ratio is 0.1:2:3, which suggests only a small amount of PhOLi was transformed into PhOH.

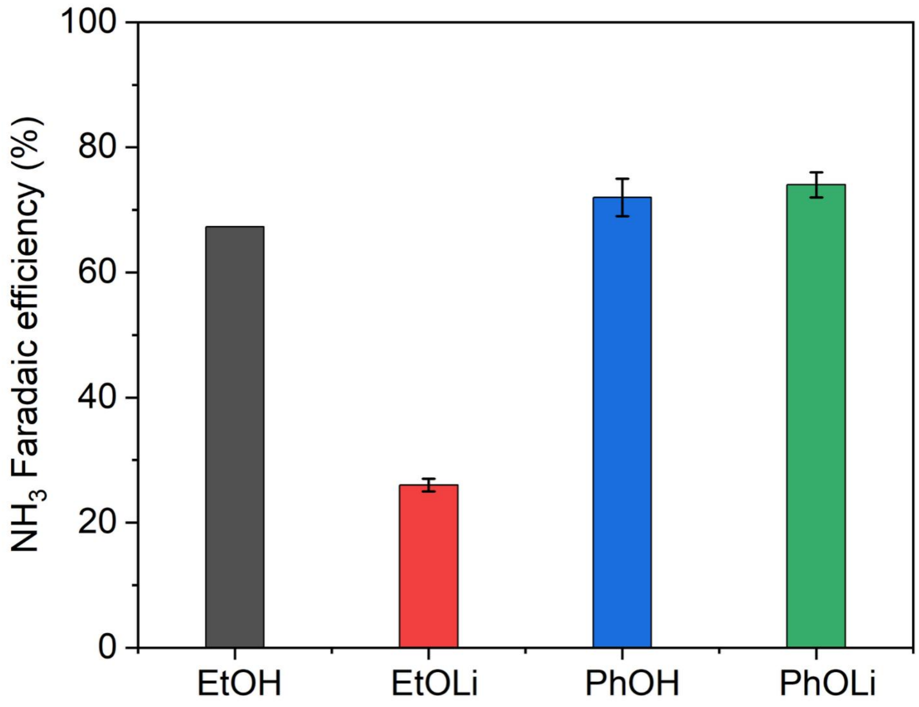

Supplementary Fig. 39. The comparison of FE between using EtOH (50 mM), EtOLi (50 mM), PhOH (37 mM), and PhOLi (37 mM) as proton shuttles under similar experimental conditions. Error bars represent the mean ± standard deviation derived from three independent measurements.

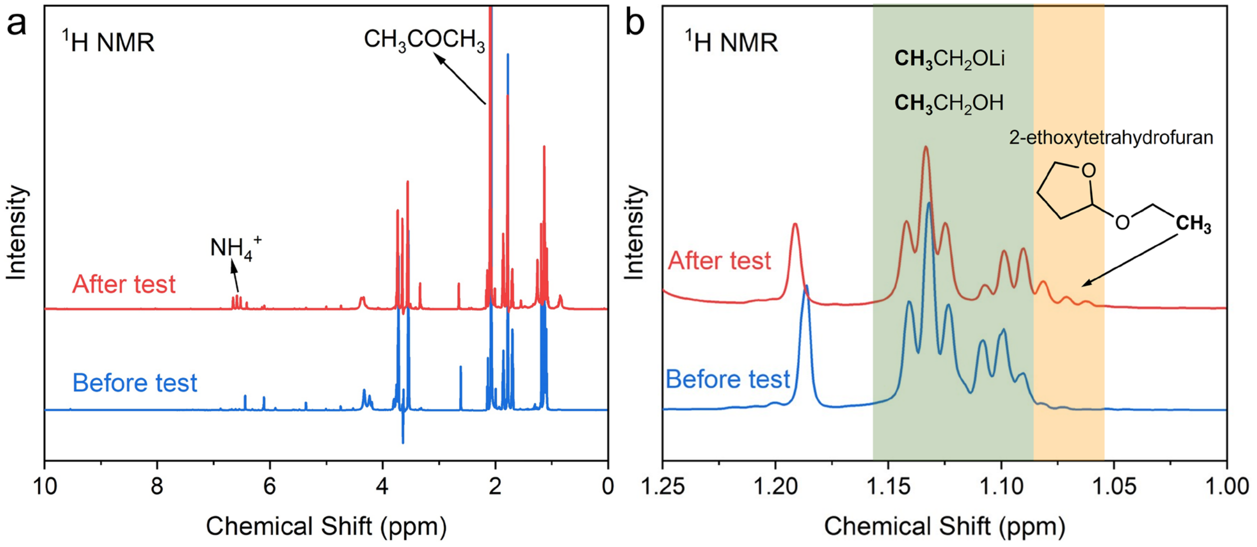

Supplementary Fig. 40. The NMR spectra of electrolytes before and after the CP test. (a) Overview of ^{1}H NMR spectra of electrolytes with EtOLi before and after the CP test. (b) A zoom-in ^{1}H NMR spectra of electrolytes with EtOLi before and after the CP test. After the electrochemical test, the new peaks at 1.6 ppm and 3.4 ppm are from the polytetrahydrofuran (polyTHF), which is a constant decomposition of the electrolyte.[1] The most plausible side product is 2-ethoxytetrahydrofuran, which is consistent with the previous report.[2]

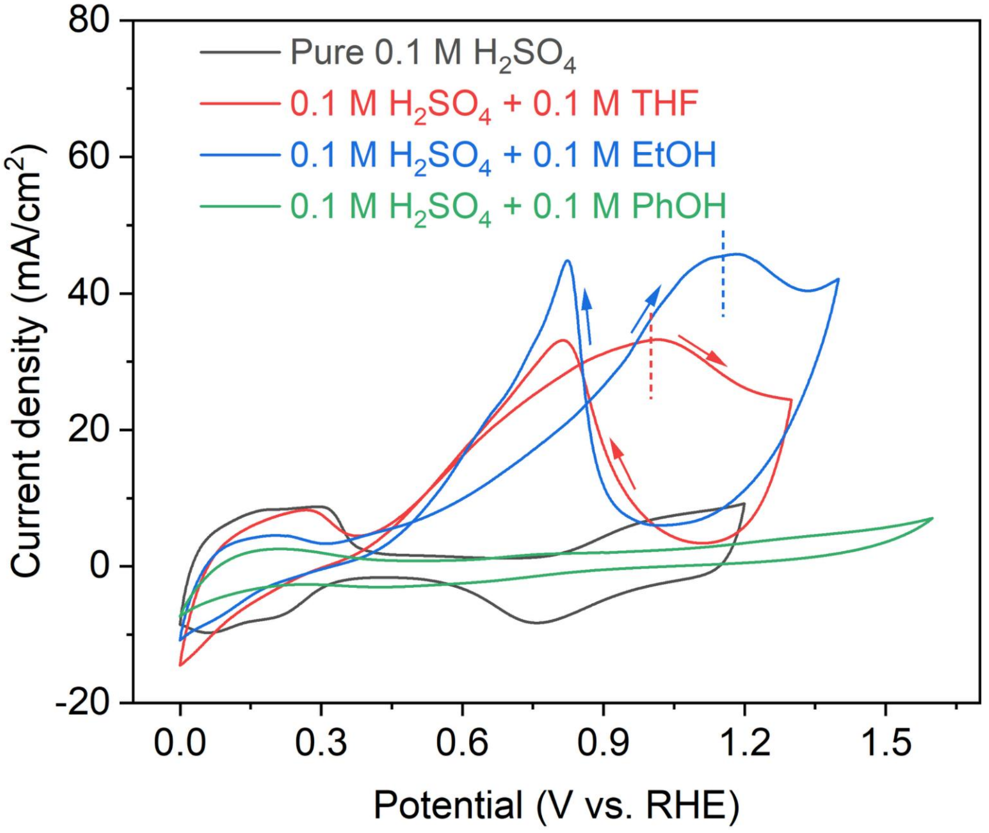

Supplementary Fig. 41. The THF, EtOH, and PhOH oxidation was investigated on PtAu/Ti stub. The benchmark CV curve was recorded between 0 and 1.2 V vs RHE at 50 mV/s in Ar-saturated 0.1 M H_2SO_4. The THF, EtOH, and PhOH oxidation activity was evaluated in Ar-saturated 0.1 M H_2SO_4 with 0.1 M THF or 0.1 M EtOH or 0.1 M PhOH electrolyte with a scan rate of 50 mV/s. The peak oxidation potential of EtOH is higher than that of THF. The oxidation current density of PhOH is much lower than that of EtOH, which indicates that PhOH exhibits much lower activity of electro-oxidation than EtOH and THF. In other words, the PhOH demonstrated superior stability than EtOH.

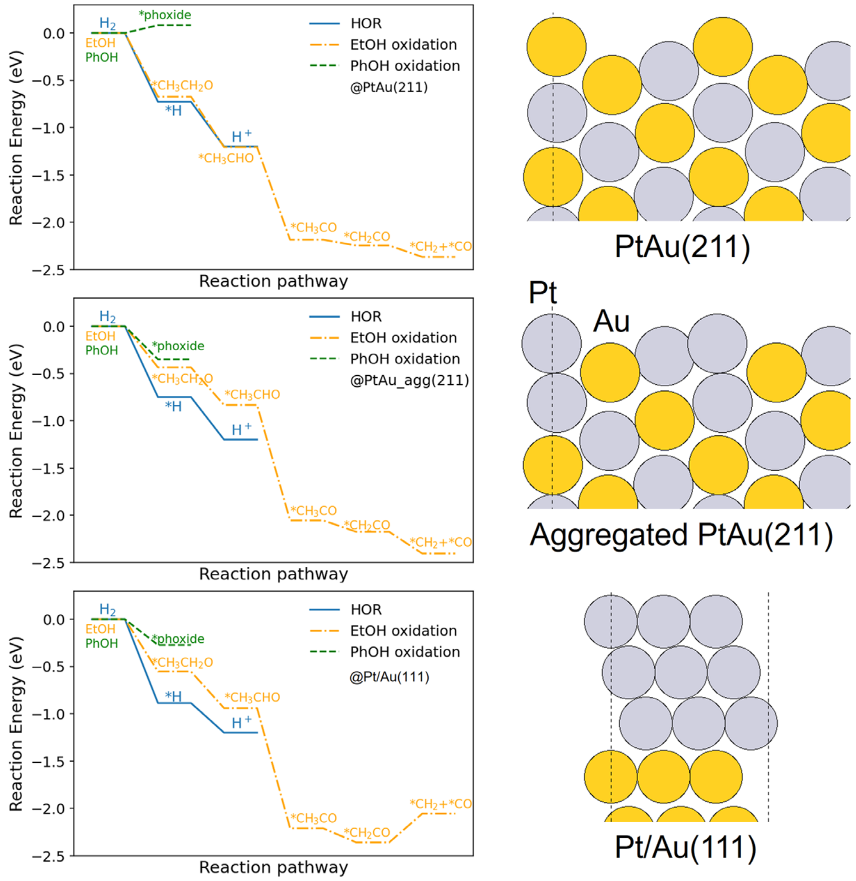

Supplementary Fig. 42. Reaction energies of HOR, ethanol oxidation, phenol oxidation on PtAu(211), aggregated PtAu(211), and Pt/Au (111) surface at 0.6 V vs. RHE.

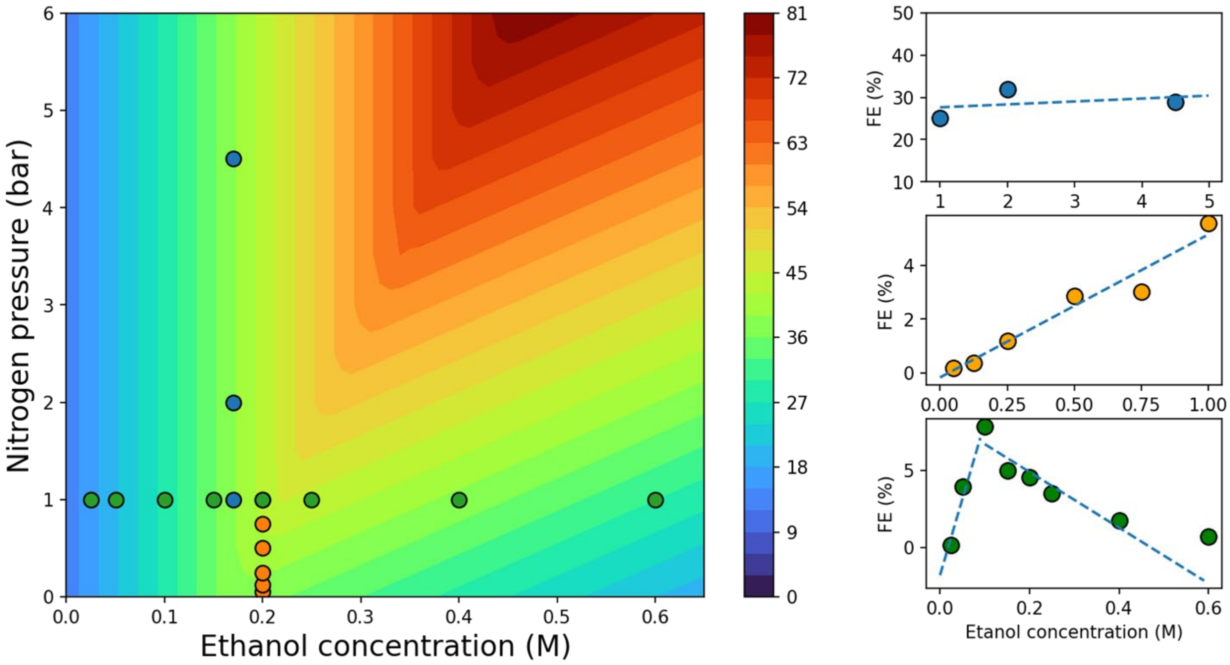

Supplementary Fig. 43. Heatmap of predicted FE as a function of N_2 pressure and ethanol concentrations. The points are collected from experimental reports[3,4].

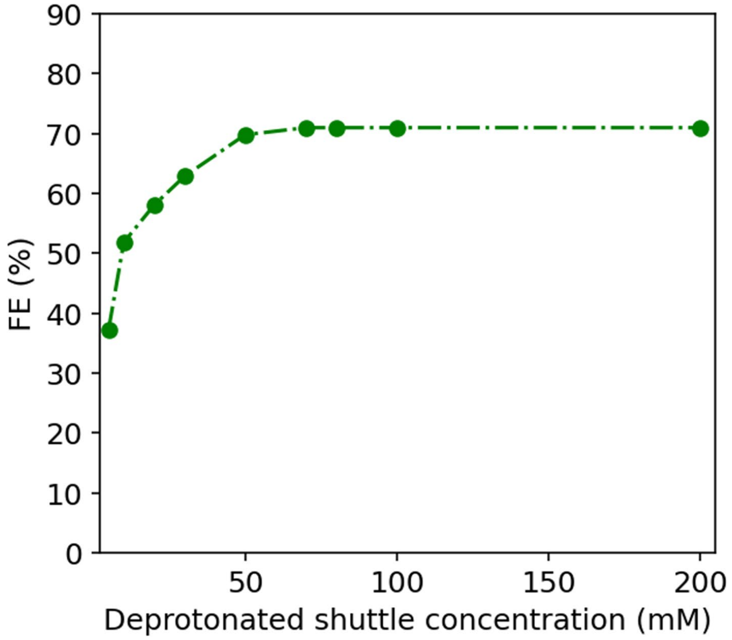

Supplementary Fig. 44. Faradaic efficiency changed with varying deprotonated shuttle (B^-) concentrations. The protonated capability of B^- was set to the same as pK_a = 30. The deprotonated form (B^-), unable to contribute to proton concentration in electrolytes, could retain the optimal performance when further increasing concentration, agreeing with experimental observation (Fig. 2e).

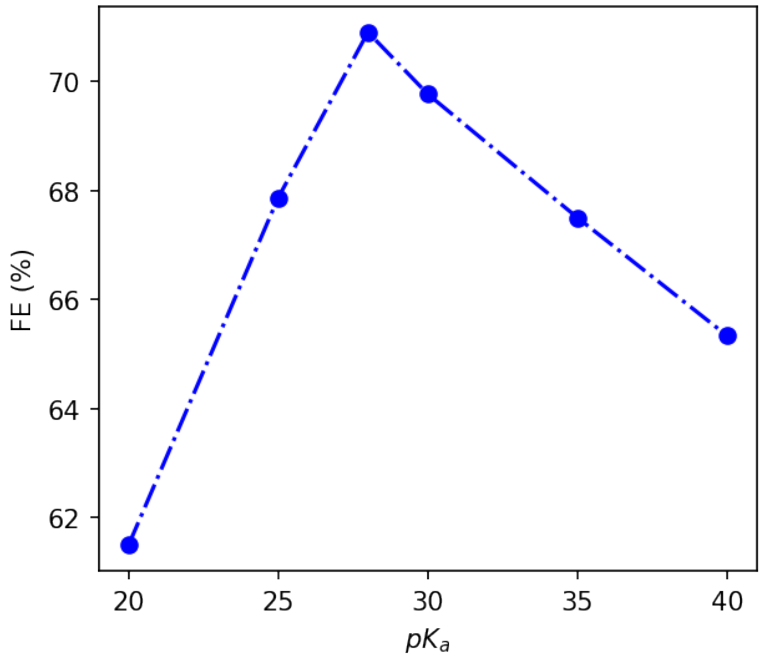

Supplementary Fig. 45. Volcano relationship between ammonia FE and pK_a of proton shuttle.

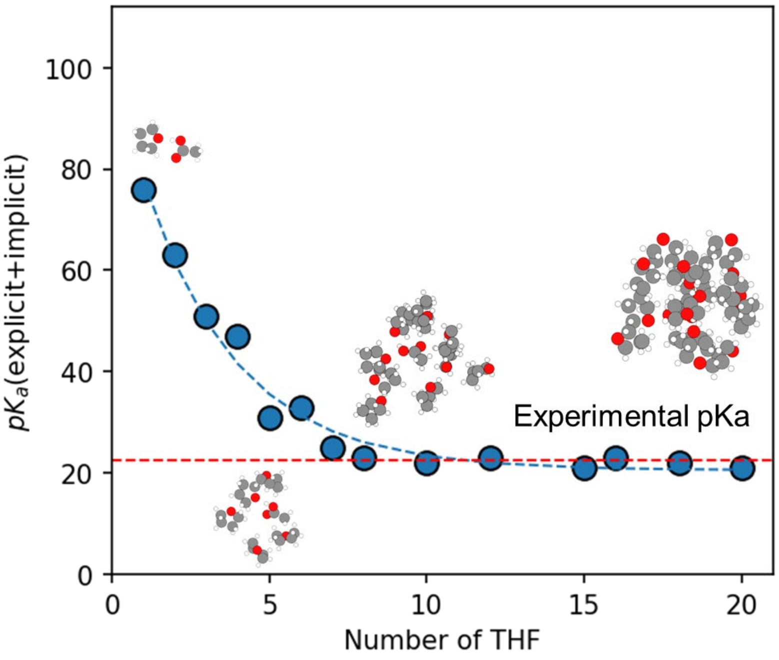

Supplementary Fig. 46. The pK_a calculations with solvent corrections, using acetic acid as the benchmark. The computational model is validated by comparing the computational pK_a value with the experimental pK_a value. The red line is the experimental pK_a value of acetic acid in the THF[5].

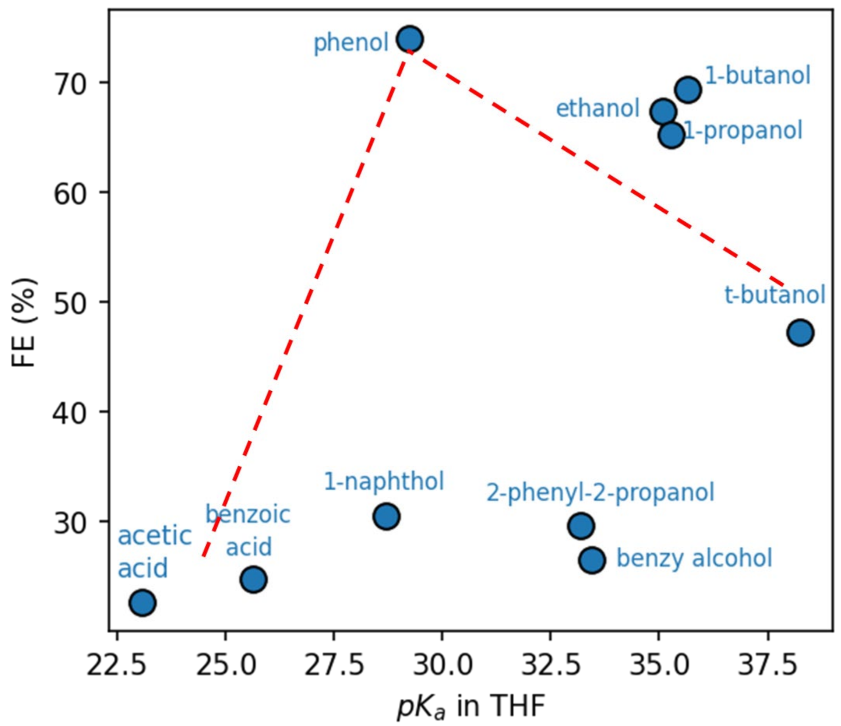

Supplementary Fig. 47. Predicted FE (line) and experimental data (points) with the single descriptor of pK$_a$.

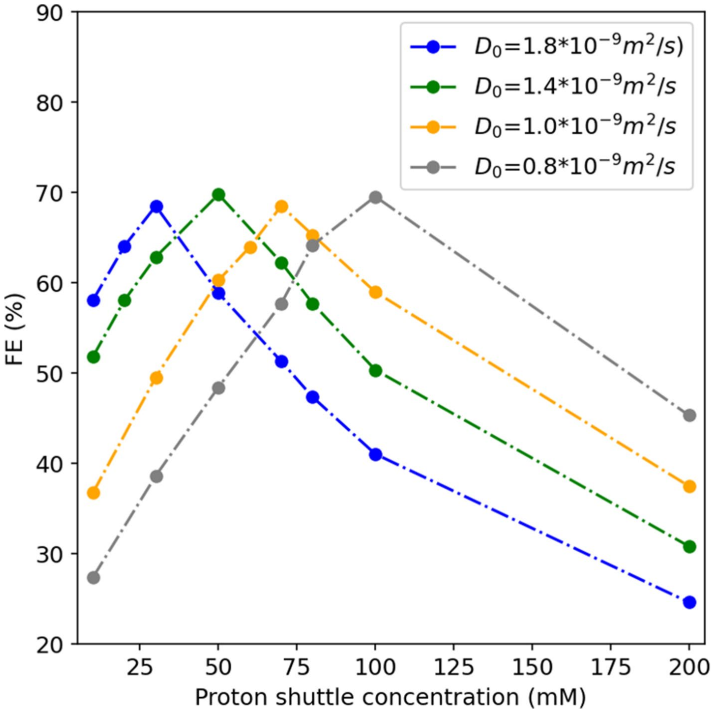

Supplementary Fig. 48. A 1D plot of ammonia faradaic efficiency versus concentrations of proton shuttle with different diffusion coefficients, at a fixed pK_a of 30. At a specific pK_a (30) value, a proton shuttle with low diffusivity would, at the same time, have slower proton diffusion, requiring compensation from increasing its concentration.

Supplementary tables

Supplementary table 1. Summary of the effect of proton shuttles on ammonia faradaic efficiency with potential cycling.

Proton shuttle	Con. (mM)	m_{NH3} (μg)	Gas-phase (μg)	Electrolyte (μg)	Electrode deposits (μg)	FE (%)
Without proton shuttle	0	11287	18	4672	6597	27.4
Phenol	25	26502	3483	21202	1817	64.4
Phenol	25	27230	5032	20372	1826	66.1
Phenol	25	26414	4715	20088	1611	64.1
Phenol	37	29449	7304	20601	1544	71.5
Phenol	37	28575	7062	20494	1019	69.4
Phenol	37	30682	7205	22195	1281	74.5
Phenol	50	25978	7989	16850	1139	63.1
Phenol	50	25810	8121	16624	1065	62.7
Phenol	50	26750	8443	16850	1457	65
Phenol	75	19953	8198	11186	569	48.5
Phenol	100	14125	6275	7201	649	34.3
Lithium phenoxide	25	30281	2017	27137	1127	73.5
Lithium phenoxide	37	29257	2246	26201	810	71.5
Lithium phenoxide	50	30237	3116	25809	1312	73.4
Lithium phenoxide	50	30074	2038	26196	1840	73
Lithium phenoxide	50	31142	3002	27372	1068	76.4
Lithium phenoxide	75	30324	3837	24141	2346	73.6
Lithium phenoxide	100	29712	4169	23865	1678	72.2
Methanol	50	15258	6505	7062	1691	37.1
Ethanol	50	27736	11682	15415	639	67.3
1-Propanol	50	26851	10256	14890	1704	65.2
1-Butanol	50	28576	11317	16272	987	69.4
Tert-butanol	50	19450	6548	11112	1790	47.2
Benzyl alcohol	50	10920	1102	7727	2091	26.5
2-Phenyl-2-propanol	50	12197	3427	6849	1921	29.6
Pyridine	50	5207	4	471	4732	12.6
DBU	50	8850	21	4598	4231	21.5
NDN	50	1854	15	978	861	4.5
Proton-sponge	50	1489	79	711	699	4
1-Naphthol	37	12565	1264	9864	1437	30.5
Hydroquinone	37	9405	147	6746	2512	22.8

Phloroglucinol	37	1663	20	663	980	4
$[P_{6,6,6,14}]$[eFAP]	50	9201	189	5593	3419	22.3
$[P_{6,6,6,14}]$[eFAP]	50	9970	215	5227	4528	24.2
$[P_{6,6,6,14}]$[eFAP]	50	10456	319	4852	5285	25.4
$[P_{6,6,6,14}]$Cl	50	5781	15	3829	1937	14.1
Acetate acid	37	9320	4030	4814	476	22.6
Acetate acid	37	9582	4179	4971	432	23.3
Acetate acid	37	8456	4527	3459	470	20.5
Benzoic acid	37	10206	1095	7718	1393	24.8
Acetate acid + PhOLi	37	12593	6120	6023	450	30.6
Acetate acid + PhOLi	37	13368	6688	5824	856	32.5
Acetate acid + PhOLi	37	12458	6656	5448	354	30.3

The working electrode is the 30 μm SSC. The electrolyte is 1 M $LiBF_4$ in THF with different ethanol concentrations. The potential cycling condition is -6 mA/cm^2 for 1 min and 0 mA/cm^2 for 1 min. The passed charge is 700 C.

Supplementary table 2. Calculated pKa of proton shuttles in THF.

Proton shuttle	Calculated pK_a value	Experimental pK_a value[5]
Acetic acid	23.02	22.48
Benzoic acid	26.02	25.11
Methanol	39.25	-
Ethanol	39.08	-
1-Propanol	39.26	-
1-Butanol	39.65	-
Tert-butanol	44.24	-
Benzyl alcohol	33.45	-
2-Phenyl-2-propanol	33.17	-
Phenol	29.24	-
1-Naphthol	29.7	-

References

1. Fu, X.*, et al.* Continuous-flow electrosynthesis of ammonia by nitrogen reduction and hydrogen oxidation. *Science*, **379**, 707-712 (2023).

2. Du, H.L.*, et al.* The chemistry of proton carriers in high-performance lithium-mediated ammonia electrosynthesis. *Energy. Environ. Sci*, **16**, 1082-1090 (2023).

3. Lazouski, N.*, et al.* Understanding Continuous Lithium-Mediated Electrochemical Nitrogen Reduction. *Joule*, **3**, 1127-1139 (2019).

4. Cherepanov, P.V.*, et al.* Understanding the Factors Determining the Faradaic Efficiency and Rate of the Lithium Redox-Mediated N_2 Reduction to Ammonia. *J. Phys. Chem. C*, **125**, 11402-11410 (2021).

5. Kutt, A.*, et al.* pK_a values in organic chemistry - Making maximum use of the available data. *Tetrahedron Lett.*, **59**, 3738-3748 (2018).
